# Mean-Square Displacements of Polymers in Simulated Blend Melts

**DOI:** 10.3390/polym17233140

**Published:** 2025-11-26

**Authors:** George D. J. Phillies

**Affiliations:** Department of Physics, Worcester Polytechnic Institute, Worcester, MA 01690, USA; phillies@4liberty.net; Tel.: +1-508-754-1859

**Keywords:** polymer dynamics, mean-square displacement, polymer melt, polymer solution, computer simulation, scaling behavior, polymer blend, scaling exponents, power-law behavior

## Abstract

We apply numerical analysis to interpret reported simulations of polymer blend melts, in particular simulational determinations of mean-square displacements g(t) of polymer beads and polymer centers of mass. Our interest is a quantitative comparison of g(t) with theoretical models that predict g(t). Many models predict that g(t) can be described as a sequence of power-law regimes $g\left(t\right)∼t^{\alpha }$. In each regime, α has a model-predicted value. We find that these models are not consistent with simulations of blend melts. Instead, g(t) generally has a single power-law regime and (when those times are reached) a long-time diffusive (α≈1) regime. Outside these two regions, if one writes $g\left(t\right)∼t^{\alpha }$, then α is a smoothly-changing function of time.

## 1. Introduction

This paper extends our prior reviews of the phenomenology of polymeric fluid dynamics. Our volume Phenomenology of Polymer Solution Dynamics [1] examined the concentration, molecular weight, and time dependences of polymer solution properties, including centrifugation, electrophoresis, polarized and depolarized light scattering spectroscopy, solvent diffusion, segmental relaxation, dielectric relaxation, mutual diffusion, probe diffusion, viscosity, viscoelasticity, and non-linear viscoelastic behavior, and the same properties as shown by solutions of spherical colloids. Comparison of experiments on polymer solutions with familiar theoretical models found that hydrodynamic scaling forms for the self-diffusion coefficient *D* as a function of polymer concentration *c* and molecular weight *M* [2], viz., $D∼\exp(-\alpha c^{\nu }M^{\gamma })$, worked well, but reptation-scaling forms [3,4], viz., $D∼c^{\nu }M^{\delta }$, are almost entirely inconsistent with experiment. The other solution transport coefficients that we studied show the same behavior, namely that they are consistent with the hydrodynamic scaling model but are not consistent with the reptation-scaling model.

When we first presented our solution dynamics results, we were challenged to discuss whether our results also applied to melts, but were not then prepared to do so. This paper is part of a series that finally responds to that challenge. There are several alternative pathways to examining the melt question. We have elected to consider the phenomenology of computer simulations. This choice has both advantages and disadvantages. On the positive side, computer simulations give exact control over the polymer molecular weight and chain topology. Computer simulations allow one to measure correlation functions that are not readily accessible to experiment, for example the amplitudes and time correlation functions of individual Rouse modes [5]. On the negative side, hardware limitations make it difficult to study high-molecular-weight polymers, because the required computation times become inaccessibly large.

There is an extended literature on simulations of polymer melts. Melt dynamic properties that have been studied would certainly include determinations of the stress modulus G(t) [6], the nonlinear viscosity $\eta (\dot{\gamma })$ [6], segmental and whole-chain orientation vector autocorrelation functions [7,8,9,10], self- (single-chain) and cross- (two-chain) correlation functions of the orientation tensor [11,12], the coherent scattering function $g^{\left(2\right)}\left(q,t\right)$ [13], the incoherent scattering function $g^{\left(1\right)}\left(q,t\right)$ [13,14,15], the torsional autocorrelation function Θ(t) [8], segmental escape from a hypothesized tube [16], and the storage and loss moduli $G^{\prime }\left(\omega \right)$ and $G^{\prime \prime }\left(\omega \right)$ [16].

We have already reviewed properties of simulated Rouse modes [17]. Of course, Rouse’s predictions [5] refer to an isolated bead-spring polymer in highly dilute solution, not to a polymer in a melt, but there are theoretical models [3,4,18,19] that invoke the Rouse model to describe polymer motions in melts on some time and distance scales, so examination of Rouse mode behavior in melts is appropriate. In an extended review [17], we demonstrated that Rouse-model predictions for Rouse mode amplitudes and their time correlation functions are generally incorrect for simulations of polymer melts. We concluded that polymer motions in the melt are not described by the Rouse model.

We then reviewed the time dependence of mean-square displacements g(t) of polymer beads and polymer centers of mass in simulated melts of monodisperse linear polymers [20,21]. There are theoretical models [3,4,18,19] that predict that the time dependence of g(t) is described as a series of power laws $g\left(t\right)∼t^{\alpha }$. These models appear to be correct in two limits: At very early times, $g\left(t\right)∼t^{2}$ corresponds to the ballistic motion predicted by Newton’s Laws of motion. At very late times, g(t)∼t corresponds to the simple diffusion predicted by Doob’s Theorem [22]. Outside these two limits, we found that power-law behavior is generally not present. In most cases, g(t) does not follow a series of power laws separated by transition regimes. Because this result was not anticipated, we made a point of surveying a large number of simulational studies, thus avoiding the suggestion that we had accidentally selected a few special cases in which power-law behavior is not encountered.

In this paper, we consider linear polymers in simulated bidisperse melts. We limit ourselves to a narrow range of dynamic properties, namely the mean-square bead displacement functions $g_{1}\left(t\right)$ and $g_{2}\left(t\right)$, and the mean-square center-of-mass mean-square displacement function $g_{3}\left(t\right)$. Rather than extending the paper indefinitely, we paused after analyzing four representative sets of results. To examine simulations of short polymers, we considered a study by Sacristan et al. [23]. To examine lightly entangled polymers, polymers with $N/N_{e}\leq 4$, we treat simulations by Kopf et al. [24]. Here *N* is the chain length and $N_{e}$ is a nominal entanglement length. Results due to Adeyemi et al., include a long polymer with $N/N_{e}>10$ [25], while results of Wang and Larson [26] extend out to chains having $N/N_{e}\approx 15$. In addition, our two previous papers [20,21] had examined simulations of bidisperse systems by Brodeck et al. [15] and by Moreno and Colmenero [27]. These studies include atomic-level simulations, united atom simulations, and invocations of the Kremer-Grest bead-spring model with various slightly different potentials. Readers are referred to the original papers for extensive details on simulation methods.

It would of course be desirable to include simulations on still-longer polymers when these become available. A further review examining g(t) of non-linear polymers, notably branched polymers and rings, is in preparation. We are aware of simulations that invoke the repton model or a slip-link model [28,29,30,31]. We did not consider those studies in this paper, because the repton and slip-link models were cleverly constructed to force polymer molecules to move via reptation, in order that the properties of a melt filled with reptating polymers could be determined. Those studies had therefore assumed an answer to the problem we are studying, namely how polymers actually move in a melt.

The remainder of this paper has three sections. The next Section presents definitions and our quantitative method for analyzing g(t) to determine its time dependence. A further Section applies our quantitative method to representative results from the simulation literature. A final Section presents our conclusions. The verisimilitude of our analysis arises from the graphical presentations of our numerical analyses, so Supplemental Figures present each of our plots at full scale.

## 2. Definitions and Methods

We first give a few definitions and note what familiar models say about them. We then describe our approach to a numerical analysis of the g(t). We have previously presented tests of our method against representative data [20].

Tube models [3,4,18,19] for polymer dynamics make a series of predictions of chain mean-square displacements, including motions of individual beads and motions of the chain center of mass. The mean-square displacement functions are the $g_{n}\left(t\right)$, where n∈(1,2,3), the $g_{n}\left(t\right)$ being defined in terms of polymer bead positions $r_{i}\left(t\right)$. Here *i* labels each of the *N* beads of a single polymer chain, while the chain center of mass is denoted $r_{\mathrm{cm}}\left(t\right)$. The average 〈…〉 includes averages over all chains and over all values of the initial time $t_{0}$.

The single-bead mean-square displacement over a time interval *t* is

(1) $$g_{1}\left(t\right)=\frac{1}{N}\sum_{i=1}^{N}\langle {\left(r_{i}\left(t+t_{0}\right)-r_{i}\left(t_{0}\right)\right)}^{2}\rangle .$$

Here $t_{0}$ and $t+t_{0}$ are times. The sum may be over the positions of all *N* beads of a chain, or over the positions of a subgroup of *n* beads in a longer chain.

The mean-square motion of the beads relative to the chain center-of-mass is

(2) $$g_{2}\left(t\right)=\frac{1}{N}\sum_{i=1}^{N}\langle {\left(\left(r_{i}\left(t+t_{0}\right)-r_{\mathrm{cm}}\left(t+t_{0}\right)\right)-\left(r_{i}\left(t_{0}\right)-r_{\mathrm{cm}}\left(t_{0}\right)\right)\right)}^{2}\rangle .$$

The corresponding center-of-mass mean-square displacement is

(3) $$g_{3}\left(t\right)=\langle {\left(r_{\mathrm{cm}}\left(t+t_{0}\right)-r_{\mathrm{cm}}\left(t_{0}\right)\right)}^{2}\rangle .$$

For linear chains, tube-reptation models predict the time dependences of these mean-square displacements, the predictions having the form of a series of power laws $g\left(t\right)∼t^{\alpha }$. The exponent α depends on the polymer chain length and the time scale under consideration. According to the tube model, the relevant time scales are separated by a segmental relaxation time $\tau_{0}$, an entanglement time $\tau_{e}$, a Rouse time $\tau_{R}$, and a disentanglement time $\tau_{d}$. Within the tube model, $\tau_{e}$ is an average time at which a diffusing polymer bead encounters a wall of the hypothesized tube. $\tau_{R}$ is the relaxation time of the longest-lived Rouse mode [5]. $\tau_{d}$ is the time at which, on the average, a polymer has diffused through its radius of gyration $R_{g}$ and therefore is said to have escaped from its tube.

For melts of chains sufficiently short that, within the *ansatz* of the tube model, the chains are unentangled, the tube model predicts for short times $t<\tau_{R}$ that chain motions are described by the relaxation of pure Rouse modes, while at long times $t>\tau_{R}$ chain motions are described by simple Brownian diffusion, leading to

(4) $$\begin{array}{ccc} g_{1}\left(t\right) & ∼ & t^{1/2}\;\mathrm{for}\;t\leq \tau_{R}, \end{array}$$

(5) $$\begin{array}{ccc} g_{1}\left(t\right) & ∼ & t\;\mathrm{for}\;t\geq \tau_{R}, \end{array}$$

and

(6) $$g_{3}\left(t\right)∼t$$

at all times.

For linear chains long enough that, according to the tube model, the chains entangle, the model predicts for $g_{1}\left(t\right)$ that

(7) $$\begin{array}{ccc} g_{1}\left(t\right) & ∼ & t\;\mathrm{for}\;t\leq \tau_{0}, \end{array}$$

(8) $$\begin{array}{ccc} g_{1}\left(t\right) & ∼ & t^{1/2}\;\mathrm{for}\;\tau_{0}\leq t\leq \tau_{e}, \end{array}$$

(9) $$\begin{array}{ccc} g_{1}\left(t\right) & ∼ & t^{1/4}\;\mathrm{for}\;\tau_{e}\leq t\leq \tau_{R}, \end{array}$$

(10) $$\begin{array}{ccc} g_{1}\left(t\right) & ∼ & t^{1/2}\;\mathrm{for}\;\tau_{R}\leq t\leq \tau_{d}, \end{array}$$

(11) $$\begin{array}{ccc} g_{1}\left(t\right) & ∼ & t\;\mathrm{for}\;\tau_{d}\leq t. \end{array}$$

For long linear chains, the tube model predicts for $g_{3}\left(t\right)$ that

(12) $$\begin{array}{ccc} g_{3}\left(t\right) & ∼ & t\;\mathrm{for}\;t\leq \tau_{e}, \end{array}$$

(13) $$\begin{array}{ccc} g_{3}\left(t\right) & ∼ & t^{1/2}\;\mathrm{for}\;\tau_{e}\leq t\leq \tau_{R}, \end{array}$$

(14) $$\begin{array}{ccc} g_{3}\left(t\right) & ∼ & t\;\mathrm{for}\;\tau_{R}\leq t. \end{array}$$

At extremely short times not treated by these models, atomic motions are expected to approach being ballistic, leading to $t^{2}$ behavior for the mean-square displacements. These predictions were originally made for monodisperse melts. However, changing the molecular weight of the matrix polymers, relative to the molecular weight of the polymer of interest, changes the importance of various mechanisms such as tube dilation and tube renewal for relaxing the chain of interest, but leaves the qualitative model including its power laws intact.

An observation of a $t^{1/4}$ regime for $g_{1}\left(t\right)$ of long chains during $\tau_{e}\leq t\leq \tau_{R}$ is sometimes said to demonstrate that the tube model is correct. However, Puetz et al. [32] note that the Schweizer mode-coupling model [33,34] predicts a $t^{0.28}$ time dependence in this regime, even though Schweizer’s description of bead motion is entirely different from the tube model.

How do we test for power-law behavior and extract values for exponents? In this paper, we represent g(t) as a generalized power law

(15) $$g\left(t\right)=g_{o}t^{\alpha (t)}.$$

Here $g_{o}$ is a constant prefactor and α(t) is a time-dependent instantaneous exponent at time *t*. Our interest is in finding the time dependence of α(t). We previously demonstrated [20] how to do this.

Our method, described below, should be contrasted—no criticism is here intended—with many literature studies in which g(t) was simply assumed, on the basis of theoretical treatments such as those described above, to have extended regions in which g(t) follows a power law in time, i.e., extended regions within which α(t) is constant. However, regions in which α is a constant were the primary focus of these studies, because in those regimes there were theoretical predictions of α. In some earlier studies, log-log plots of g(t) against *t* were to be compared with lines drawn to guide the eye, the lines sometimes being placed so that they did not overlay the data. In other studies, linear fits were made of sections of log(g(t)) to a form $\log\left(g_{o}\right)+\alpha \log\left(t\right)$, with α fixed in advance or used as a fitting parameter. In all these cases, simple power-law behavior of g(t) was assumed. Note, however, a paper by Nahali and Rosa [35], who obtained from determinations of g(t) a time-dependent series of values of α(t), using a method for determining α(t) that is apparently not the same as ours.

Our objective here is to test whether power-law behavior is actually present. Determinations of power-law exponents must wait until the presence of power-law behavior is established. Our approach is to take published simulations of $g_{n}\left(t\right)$, and extract from them the logarithmic derivative $\alpha \left(t\right)=d\log(g_{n}\left(t\right))/d\log\left(t\right)$, which is the local value of α(t) in $g\left(t\right)∼t^{\alpha (t)}$.

Slight caution is needed in extracting the $g_{n}\left(t\right)$ from the literature. While many authors simply computed the $g_{n}\left(t\right)$ from the record of the bead positions, a few authors first calculated the incoherent scattering function $g^{\left(1\right)}\left(t\right)$ as

(16) $$g^{\left(1\right)}\left(t\right)=\sum_{i}\langle \exp\left(-ıq\cdot \left(r_{i}\left(\tau \right)-r_{i}\left(\tau +t\right)\right)\right)\rangle ,$$

where here the sum is over all scatterers, q is a scattering vector, and 〈…〉 denotes an average. They then applied a supposed Gaussian approximation

(17) $$g^{\left(1\right)}\left(t\right)=\exp\left(-\frac{1}{2}q^{2}\langle {\left(\Delta x\left(t\right)\right)}^{2}\rangle \right)$$

to extract from $g^{\left(1\right)}\left(t\right)$ a nominal mean-square displacement $\langle {\left(\Delta x\left(t\right)\right)}^{2}\rangle$. However, if the Gaussian approximation is correct as arising from assumed uncorrelated forces on each polymer chain, then Doob’s Theorem [22] guarantees that $\langle {\left(\Delta x\left(t\right)\right)}^{2}\rangle$ increases linearly in time and $g^{\left(1\right)}\left(t\right)$ necessarily has a simple exponential form. If, on the other hand, as is almost always the case, $g^{\left(1\right)}\left(t\right)$ is not a simple exponential, then Equation (17) is inapplicable to the problem, and any $\langle {\left(\Delta x\left(t\right)\right)}^{2}\rangle$ nominally extracted from $g^{\left(1\right)}\left(t\right)$ via Equation (17) will be incorrect, in some cases by an order of magnitude or more. See ref. [36] for an extended analysis of some of the errors that arise from invoking the Gaussian approximation when it does not apply.

Our approach to extracting an exponent α(t) begins with a plot of log(g(t)) against log(t). As an approximation, we fit log(g(t)) to a smooth curve

(18) $$\log\left(g\left(t\right)\right)=\sum_{n=0}^{N_{t}}a_{n}{\left(\log\left(t\right)\right)}^{n},$$

where the $a_{n}$ are coefficients obtained from a linear-least-mean-squares fit of Equation (18) to the measured g(t), and where $N_{t}$ is a truncation limit.

As we previously demonstrated [20], with modest values for $N_{t}$ Equation (18)—which may be recognized as a Taylor series expansion in log(t)—accommodates well both to regions where log(g(t)) is linear in log(t) and to regions where the slope α(t) of log(g(t)) depends non-trivially on *t*. How accurate are our fits? An unknowable error is introduced when we digitized literature reports of mean-square displacements—almost always presented as graphs. The accuracy of our fits may be judged from the figures in the Supplemental Material. The fitted curves are the solid lines; sampled points from the original simulations are small cicles. In most cases, the fitted curves pass through the center of the circles, showing that the fitted curves describe the simulation results with no visible error.

The instantaneous value of α(t) follows from Equation (18) via

(19) α(t)=dlog(g(t))/dlog(t).

For the g(t) we recovered from the literature, fits with $N_{t}$ in the range 6–8 almost always gave curves that agreed with the reported g(t). We display our results graphically, comparing reported measurements of g(t) to our fitted curve and to the corresponding values for α(t).

There are two practical restrictions. First, the curves generated from Equation (18) are interpolants, not extrapolants. Extrapolating a fitted curve to times outside the range over which g(t) is reported may give unphysical results. In particular, as t→∞ our polynomial will always diverge to ±∞. A representative example of an unphysical extrapolation is discussed below. with discussion there. Second, the fitting process is sensitive to statistical noise near the end points of the measured g(t). At an end point, the fit is somewhat free to change its slope to accommodate a final few points that, due to statistical fluctuations, are too high or too low, without the change in slope creating excessive root-mean-square error from hypothetical times beyond the end points.

## 3. Simulations of Blends

We now turn to a series of simulations that determined polymer mean-square displacements in melts of polymer blends. We show results of Sacristan et al. [23] Kopf, et al. [24] Wang and Larson, [26] and Adeyemi et al. [25]. These authors treated systems that, in terms of the tube model, are unentangled, lightly entangled, or moderately entangled.

Sacristan et al. [23] report simulations of a 20 wt% polyethylene oxide: 80 wt% polymethylmethacrylate blend and a polyethylene oxide:polymethylmethacrylate block copolymer having the same 20:80 weight composition for the two blocks. Their interest was the effect of the local composition of the system around each polymer bead. By ‘local composition’, they refer to the number of beads of each monomer species in the immediate vicinity, 5–25 Å, of the bead of interest. The effective concentration of each species around a bead of interest differs between the blend and the block copolymer because the block copolymer has intramolecular connectivity that forces some beads of each species to remain nearly adjacent to each other. These simulations are of interest here because the polymers are both short, namely 30 monomers for the PEO and 10 monomers for the PMMA. Entanglement issues should therefore be minimal. The polymers were represented with a united atom formalism, using a force field taken from the literature [37,38]. The authors calculated intermolecular pair correlation functions, local concentrations of monomers as functions of distance from a given monomer, mean-square displacements $g_{1}\left(t\right)$ and $g_{3}\left(t\right)$, and the self intermediate scattering function $g^{\left(1\right)}\left(q,t\right)$. $g^{\left(1\right)}\left(q,t\right)$ was found to be bimodal; Sacristan et al., demonstrate that its slow relaxation is described by a stretched exponential in time.

**Figure 1:**
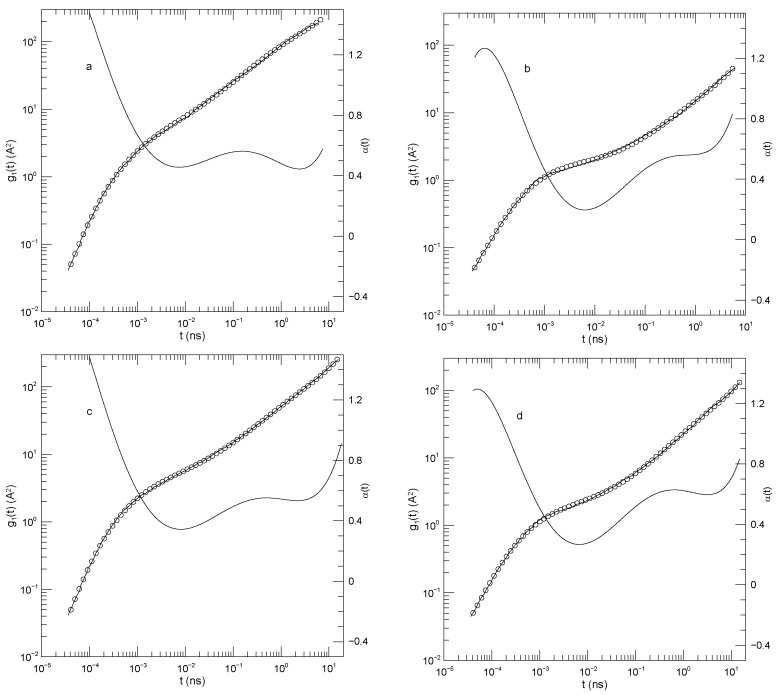
Mean-square center-of-mass displacements $g_{1}\left(t\right)$ (thick lines), our fits of $g_{1}\left(t\right)$ to eighth-order polynomials (circles), and the corresponding first logarithmic derivatives α(t) (thin lines). The subfigures show (**a**) polyethylene oxide chains in a polyethylene oxide-polymethylmethacrylate blend, (**b**) polymethylmethacrylate chains in a polyethylene oxide-polymethylmethacrylate blend, (**c**) the polyethylene oxide segment of a polyethylene oxide-polymethylmethacrylate diblock copolymer, and (**d**) the polymethylmethacrylate segment of a polyethylene oxide-polymethylmethacrylate diblock copolymer, based on simulations by Sacristan et al. [23].

In summary, Sacristan et al.’s [23] results for the mean-square displacements of their short polymers, as described in detail below, find that the local exponent α(t) that describes dlog(g(t))/dlog(t) is initially large, decreases to a single local minimum, increases to at most one plateau, and then increases again, a long-time limiting slope not being reached over the times studied in these simulations. The plateau is least evident for the motions of polyethylene oxide in the blend, as seen in Figure 1a. Said differently, in these measurements g(t) is found to have at most one power-law regime, that being seen at intermediate times.

Figure 1 shows Sacristan et al.’s [23] measurements of $g_{1}\left(t\right)$ for the two polymers in the blend and for the PEO and PMMA segments of the copolymer. In computing mean-square bead displacements of the two diblock components, Sacristan et al., [23] did not include a few monomers near the junction between the two diblock components. Transforming from the blend to the diblock copolymer slows the faster-moving PEO chains and speeds the slower-moving PMMA chains. At sufficiently long times the mean-square displacements of the PEO and PMMA segments of the diblock copolymer must converge, but Sacristan et al., [23] did not reach such extended times. The qualitative behavior of $g_{1}\left(t\right)$ is the same for both types of monomer, either in the blend or the diblock: In all cases, α(t) is initially quite large, at least 1.5 for the blend and 1.28±0.01 for the two chains of the diblock. α(t) then has a local minimum near $1\times 10^{-2}$ nS, following which it increases again. At times in the range 0.5–5 nS, α(t) approaches a plateau, the plateau being least flat for the polyethylene oxide chains in the blend, but quite nearly constant in the other three subfigures, with α(t) close to 0.53 or 0.63. At times beyond a few ns, α(t) increases again.

The depth of the first minimum varies substantially over the four subfigures. These are all clear minima, not regimes where α(t) is a constant. In the blend, the first minimum is at α(t)=0.46 for the PEO monomers and at α(t)=0.2 for the PMMA monomers. For the diblock copolymers, α(t) has a minimum at α(t)=0.34 for the PEO chain segment and α(t)=0.27 for the PMMA chain segment.

**Figure 2:**
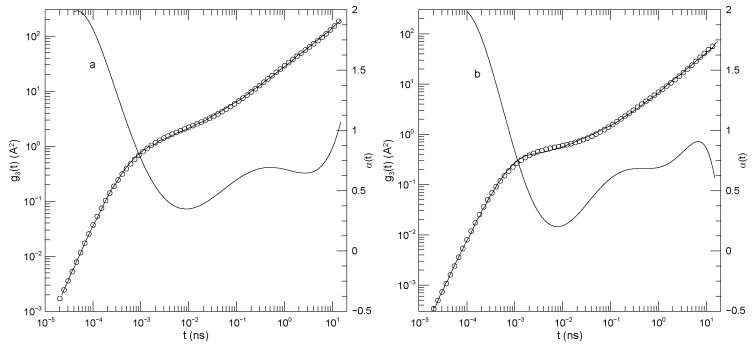
Mean-square center-of-mass displacements $g_{3}\left(t\right)$ (thick lines), our fits of $g_{3}\left(t\right)$ to eighth-order polynomials (circles), and the corresponding first logarithmic derivatives α(t) (thin lines). The subfigures show (**a**) polyethylene oxide chains in a polyethylene oxide-polymethylmethacrylate blend and (**b**) a polyethylene oxide:polymethylmethacrylate block copolymer, based on simulations of Sacristan et al. [23]. Mean-square displacements of polymethylmethacrylate in the blend are nearly equal to mean-square center-of-mass displacements of the diblock copolymers, so they are not shown separately.

Figure 2 shows Sacristan et al.’s [23] measurements for center-of-mass mean-square displacements. In both subfigures, α(t) at early times is ≈2.0. α(t) then declines to a minimum at times near $10^{-2}$ nS. The minimum is at α(t)=0.35 for the PEO, but α(t)=0.2 for the copolymer. At still larger times, α(t) increases, reaching a near-plateau having α≈2/3. That value for α is not a prediction of any of the standard theoretical models. The long-time increase to α(t)≈1 is finally approached.

Kopf et al. [24] used the Kremer-Grest model [39] for polymer dynamics to simulate a bidisperse system of polymers. Their interest was in studying properties of blends of slow- and fast-moving polymers, the two polymeric species other than their relative speeds being as similar as possible. Kopf et al. [24] obtained polymers differing only in the speed of their motions by considering a blend of two polymer species, the species differing only in the masses of their individual beads. Beads of the fast species were assigned nominal mass m=1. In different simulations, beads of the slow species had masses 1, 4, or 100, the heavier beads leading to less rapid polymer motions. Other than the bead mass, the two polymer species were identical. Chain lengths in different systems ranged from 10 to 150 beads, the entanglement length being $N_{e}\approx 33$ beads. The simulation box, a cube with periodic boundary conditions, contained between 16 and 30 polymer chains. To confirm that their results agreed with Kremer and Grest’s model, Kopf et al. [24] computed static properties including the mean-square end-to-end distance, the radius of gyration, and the single-chain static structure factor, finding agreement with Kremer and Grest in all cases.

Kopf et al. emphasize results on 20- and 30-bead polymers, these lengths being chosen to be short enough that entanglement effects are not significant but long enough that chain statistics show random-walk behavior. The study included properties of pure melts of each of their four species, and blends of the m=1 polymer with a heavy polymer at mole ratios of 80:20, 50:50, and 20:80. They compute $g_{1}\left(t\right)$ from the motions of a single bead located at the midpoint of the chain. Kopf et al. [24] divide their results into three time regimes, namely a short time regime with ’deterministic’ motion, an intermediate time regime said to be described by Rouse dynamics, and a long-time regime approximated by free diffusion.

**Figure 3:**
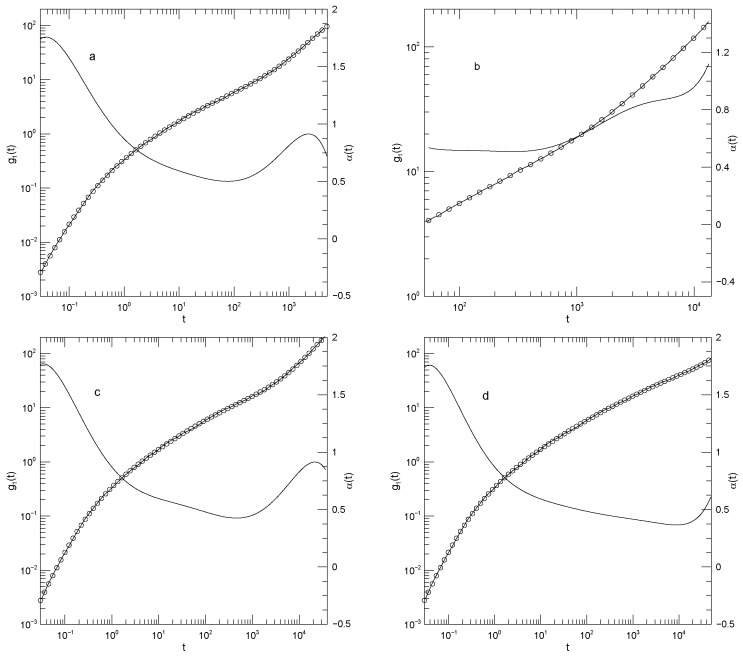
Mean-square central bead displacements $g_{1}\left(t\right)$ (thick lines) of melts of Kremer-Grest bead-spring chains, based on simulations of Kopf et al. [24], together with fits to eighth-order polynomials (circles), and the corresponding first logarithmic derivatives α(t) (thin lines). Chains contained (**a**) 20, (**b**) 30, (**c**) 50, or (**d**) 150 beads.

**Figure 4:**
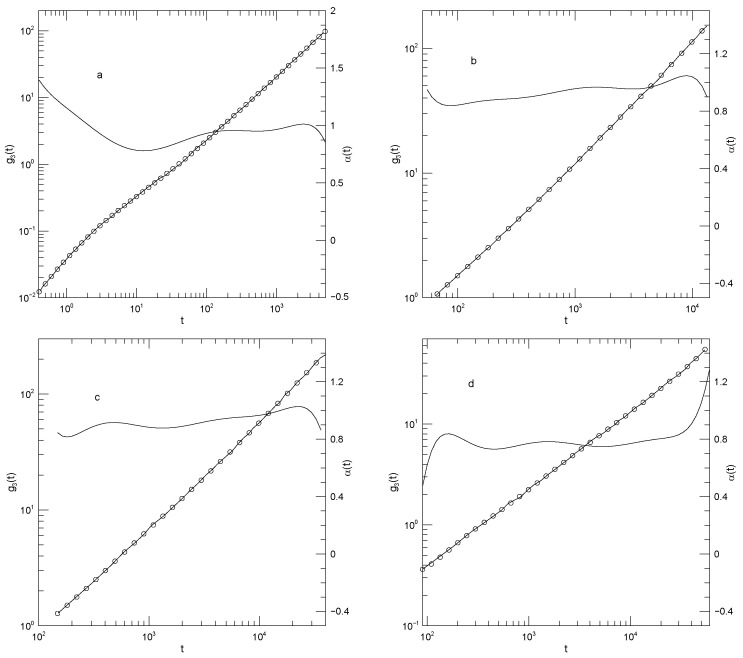
Mean-square center-of-mass displacements $g_{3}\left(t\right)$ (thick lines) of melts of Kremer-Grest bead- spring chains, based on simulations of Kopf et al. [24], together with fits to eighth-order polynomials (circles), and the corresponding first logarithmic derivatives α(t) (thin lines). Chains contained (**a**) 20, (**b**) 30, (**c**) 50, or (**d**) 150 beads.

Figure 3 and Figure 4 show $g_{1}\left(t\right)$ and $g_{3}\left(t\right)$, respectively, for monodisperse polymers having chain lengths of 20, 30, 50, or 150 beads. The 30-bead chains were only reported for longer times. As seen in Figure 3, $g_{1}\left(t\right)$ is independent of chain length for $t≲10^{2}$. At longer times, the mean-square bead displacement slows as chain length is increased. At short times t≈0.03, α(t) is ≈1.75. For the 20-, 50-, and 150-bead polymers, as time increases α(t) falls to a single minimum and then increases again. The 30-bead polymer was only examined over a narrow range of times, but it did have an apparent minimum. The minimum in α(t) decreased with increasing polymer length, being 0.50, 0.51, 0.43, or 0.37, respectively, for the four chain lengths. The minima clearly represent inflection points of $g_{1}\left(t\right)$. Power-law behavior, a region where α(t) is constant, was approximately present for the 30-bead polymer at times $t<10^{3}$ but was absent for the other three polymers.

In contrast to $g_{1}\left(t\right)$, the mean-square center-of-mass motion shown by $g_{3}\left(t\right)$, as seen in Figure 4, sometimes showed power-law behavior. For long times $t≳10^{2}$, the three shorter polymers have α(t)≈1, corresponding to simple diffusion. Over the same time period, the 150-bead polymer also shows close-to-power-law behavior with α(t)≈0.8, i.e., $g_{3}\left(t\right)∼t^{0.8}$. This exponent is not familiar from standard models.

**Figure 5:**
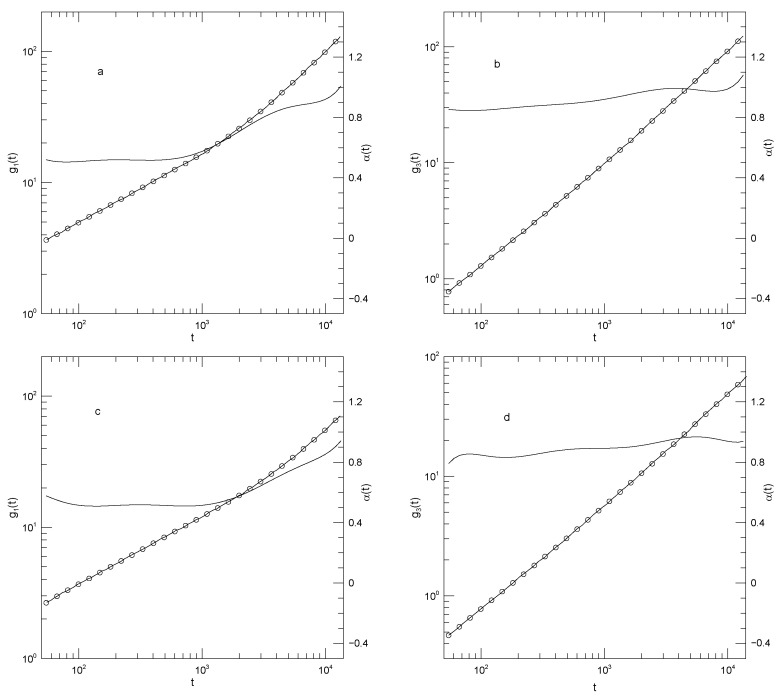
Mean-square displacements (thick lines) of the light chains in a 50:50 light-heavy polymer blend of Kremer-Grest bead-spring chains, based on simulations of Kopf et al. [24], together with fits to eighth-order polynomials (circles), and the corresponding first logarithmic derivatives α(t) (thin lines). All chains had N=30; the light chains had m=1. Figures show (**a**,**c**) $g_{1}\left(t\right)$, (**b**,**d**) $g_{3}\left(t\right)$, (**a**,**b**) m=4 heavy chains, and (**c**,**d**) m=100 heavy chains.

**Figure 6:**
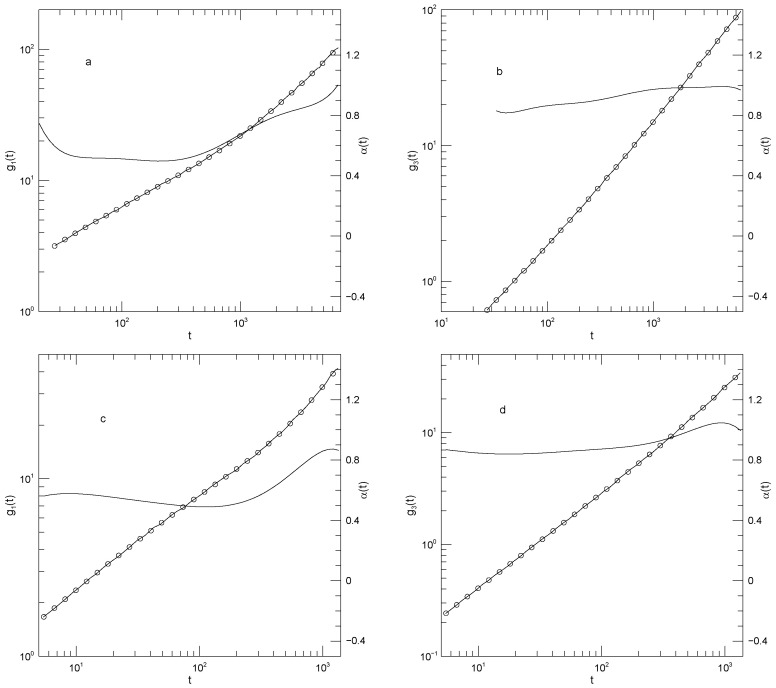
Mean-square displacements (thick lines) of the heavy chains in a 50:50 light-heavy polymer blend of Kremer-Grest bead-spring chains, based on simulations of Kopf et al. [24], together with fits to eighth-order polynomials (circles), and the corresponding first logarithmic derivatives α(t) (thin lines). All chains had N=30; the light chains had m=1. Figures show (**a**,**c**) $g_{1}\left(t\right)$, (**b**,**d**) $g_{3}\left(t\right)$, (**a**,**b**) m=4 heavy chains, and (**c**,**d**) m=100 heavy chains.

Figure 5 and Figure 6 show $g_{1}\left(t\right)$ and $g_{3}\left(t\right)$ for 30-bead polymers in 50:50 blends, the beads of the second species in the blend having mass 4 or mass 100. The effect on the motions of the lighter chains of changing the mass of the heavier polymer’s beads is modest. Figure 5 refers to motions of the light (m=1) chains through the blend, while Figure 6 refers to motions of the heavier (m=4 or m=100) chains through the blend. In the blends, $g_{1}\left(t\right)$ and $g_{3}\left(t\right)$ of the lighter chains both show substantial power-law regimes. For $g_{1}\left(t\right)$ of the lighter chains, regardless of the mass of the heavy chains, α(t) at short times is a near-constant ≈0.5, while at later times α(t) appears to climb toward 0.9. For $g_{3}\left(t\right)$ of the lighter chains, regardless of the mass of the heavy chains, α(t) increases from 0.85 at earlier times to 0.95 or 1.0 at later times.

The motions of the heavier chains (m=4 or m=100), as seen in Figure 6, are quite similar to those of the lighter chains. Changing the mass of the heavier chains has little qualitative effect on their dynamics, perhaps because the systems are heavily overdamped, so that chain inertia is negligible. α(t) from $g_{1}\left(t\right)$ of the heavier chains is ≈0.5 at earlier times. At later times it increases toward 0.9 or 1.0. α(t) from $g_{3}\left(t\right)$ of the heavier chains is not quite constant. It increases from 0.83±0.01 at the shortest time observed to 1.0 at long times. In the m=4 blend, at long times α(t) from $g_{3}\left(t\right)$ is indeed constant, showing long-time simple diffusive behavior.

**Figure 7:**
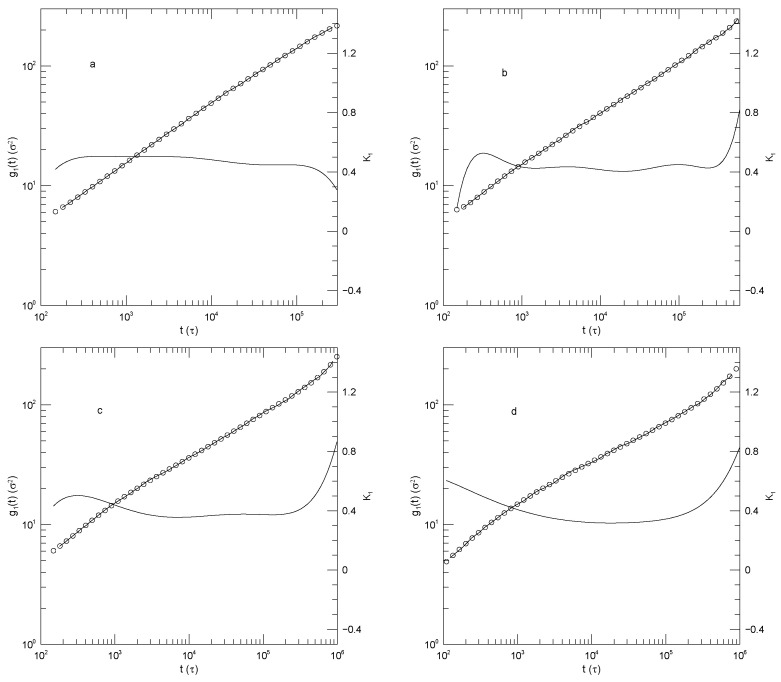
Mean-square single-bead displacements $g_{1}\left(t\right)$ (thick lines) of 350-bead Kremer-Grest bead-spring chains dissolved in melts of (**a**) 25-, (**b**) 50-, (**c**) 80-, or (**d**) 160-bead bead-spring polymers, based on simulations of Wang and Larson [26], together with fits to eighth-order polynomials (circles), and the corresponding first logarithmic derivatives α(t) (thin lines).

**Figure 8:**
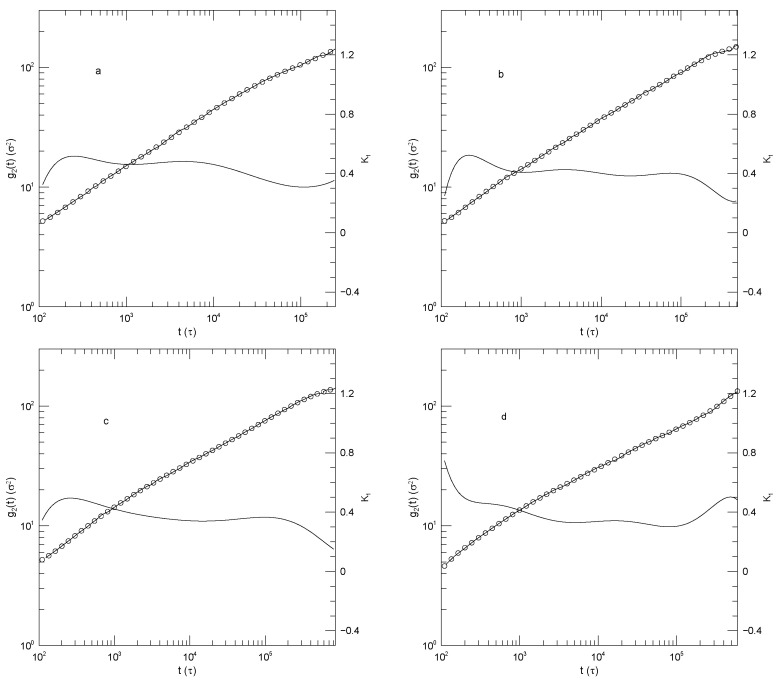
Mean-square single-bead displacements relative to chain centers of mass $g_{2}\left(t\right)$ (thick lines) of 350-bead Kremer-Grest bead-spring chains dissolved in melts of (**a**) 25-, (**b**) 50-, (**c**) 80-, or (**d**) 160-bead bead-spring polymers, based on simulations of Wang and Larson [26], together with fits to eighth-order polynomials (circles), and the corresponding first logarithmic derivatives α(t) (thin lines).

**Figure 9:**
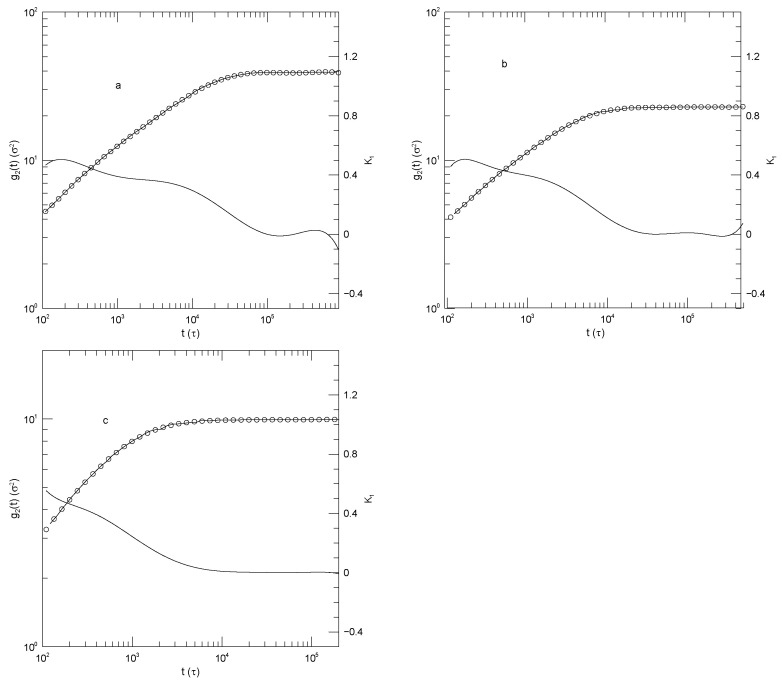
Mean-square single-bead displacements relative to chain centers of mass $g_{2}\left(t\right)$ (thick lines) of (**a**) 80-, (**b**) 50-, and (**c**) 25-bead bead-spring Kremer-Grest bead-spring polymers in their own melts, each diluted with ϕ=0.15 volume fraction of a 350-bead polymer having the same potential energy parameters, based on simulations of Wang and Larson [26]. The figures also show fits to eighth-order polynomials (circles), and the corresponding first logarithmic derivatives α(t) (thin lines).

**Figure 10:**
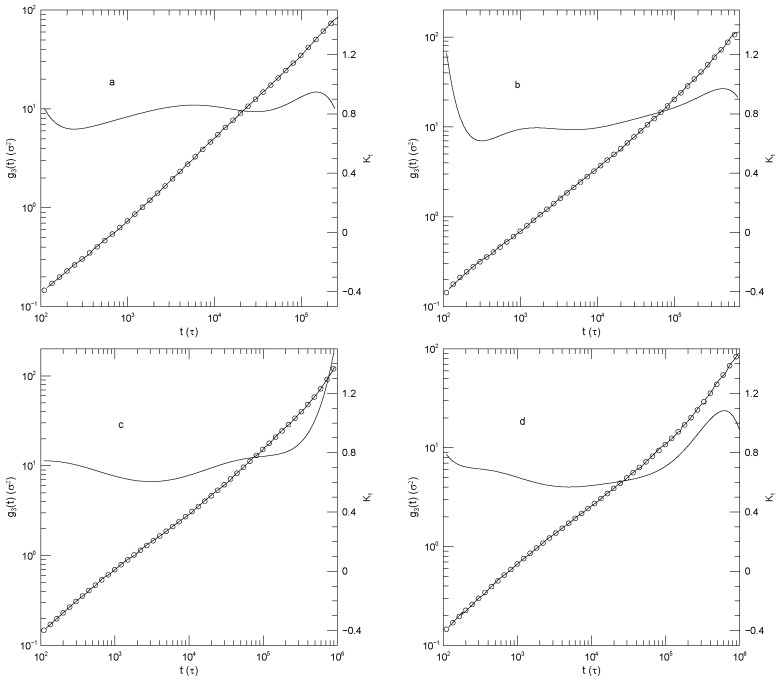
Mean-square center-of-mass displacements $g_{3}\left(t\right)$ (thick lines) of 350-bead Kremer-Grest bead-spring chains dissolved in melts of (**a**) 25-, (**b**) 50-, (**c**) 80-, or (**d**) 160-bead bead-spring polymers, based on simulations of Wang and Larson [26], together with fits to eighth-order polynomials (circles), and the corresponding first logarithmic derivatives α(t) (thin lines).

**Figure 11:**
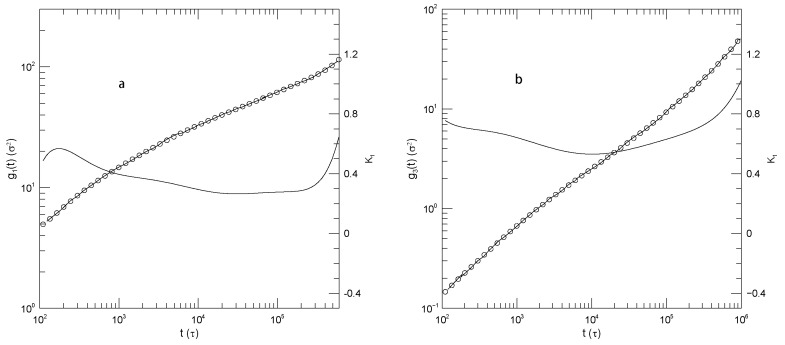
Mean-square displacements (**a**) $g_{1}\left(t\right)$ and (**b**) $g_{3}\left(t\right)$ (thick lines) of monodisperse melts of 350-bead Kremer-Grest bead-spring chains, based on simulations of Wang and Larson [26], together with fits to eighth-order polynomials (circles), and the corresponding first logarithmic derivatives α(t) (thin lines).

Wang and Larson [26] simulated the motions of dilute long-chain ($N_{L}=350$) polymers through matrices of shorter polymers ($N_{S}\in \left(25,160\right)$). Their objective was to study the effect of constraint release in the diffusion of the 350-bead chains, using the rationale that the importance of constraint release increases as the length of the matrix chains is reduced. They report $g_{1}\left(t\right)$, $g_{2}\left(t\right)$ and $g_{3}\left(t\right)$ for the long chains, $g_{2}\left(t\right)$ for the short chains, a chain diffusion coefficient *D*, and an estimate of the distribution of lifespans during which short chains remain within the nominal tube surrounding a given chain. The volume fraction of the long chains was ϕ=0.15; increasing the volume fraction of the long chains to 0.2 had nearly no effect on the long-chain dynamics. The entanglement length $N_{e}$ was estimated as 23 based on primitive path analysis and as 32 as calculated from the tube diameter as inferred from the measured single-chain dynamic structure factor S(k,t). $N_{e}\approx 32$ is in good agreement with the $N_{e}\approx 33$ inferred by Adeyemi et al. [25] from their determinations (see below) of $g_{1}\left(t\right)$. Wang and Larson’s results and our analyses are seen in Figure 7, Figure 8, Figure 9, Figure 10 and Figure 11.

Figure 7 shows mean-square single-bead displacements of the 350-bead polymer in each of the four matrix polymers. In the 25-bead polymer matrix, $g_{1}\left(t\right)$ is close to a power law, with α(t) in the range 0.51–0.44. In the 50- and 100-bead polymer matrices, α(t) has a local maximum in the range 0.50–0.53. There is then an extended region in which α(t) is close to constant, namely near 0.43 in the 50-bead polymer and 0.37 in the 80-bead polymer. For 350-bead chains in the 160-bead polymer matrix, α(t) first decreases from 0.60 to 0.32, and then increases to >0.8. The α(t)≈0.32 minimum appears to be a broad saddle point, not a power-law region.

Figure 8 shows $g_{2}\left(t\right)$ for the 350-bead polymer in the four matrices. In all four matrices, at early times α(t)≈0.5 is a local maximum. α(t) then decreases. There is then a single power-law region, with α(t) of 0.46 in the 25-bead polymer and 0.40, 0.35, and ≈0.30, respectively, in the three longer-chain matrices. In the 25-bead matrix, α(t) has a final local minimum at α(t)=0.31. It is expected for $g_{2}\left(t\right)$ that α(t) must necessarily go to zero at sufficiently long times, but for the 350-bead polymer those times were not reached in this study.

Figure 9 shows $g_{2}\left(t\right)$ for the three shorter polymers. In all three cases, α(t) decreases smoothly to zero at longer times and remains there, as expected. The initial maximum is 0.50 for the two longer polymers and 0.58 for the shortest polymer. The only power-law regime here is the long-time $t^{0}$ regime.

Figure 10 shows $g_{3}\left(t\right)$ for the 350-bead polymer in each of the four matrix polymers. The time dependence of α(t) is qualitatively the same for all four matrices. There is an early minimum in α(t), and hence a saddle point in $g_{3}\left(t\right)$. With increasing matrix polymer molecular weight, the saddle point moves to later times, and also deepens, from α(t)=0.70 in the 25-bead matrix to 0.57 in the 160-bead matrix. With two of the three shorter matrix chains, at times after the saddle point there is a single region that could be described as a power law in $g_{3}\left(t\right)$ (i.e., α(t) has a single region where α(t) is approximately constant), following which α(t) appears to increase toward the free-diffusion limit α(t)=1.

Figure 11 shows $g_{1}\left(t\right)$ and $g_{3}\left(t\right)$ for the 350-bead polymer in its monodisperse melt. $g_{1}\left(t\right)$ has a single power-law region, namely α(t)≈0.27 at late times. At earlier and later times, α(t) is larger, rising to ≈0.52 at early times and ≈0.6 at the longest times studied. $g_{3}\left(t\right)$ clearly does not have a power-law region. Instead, its α(t) decreases from 0.7 at early times to a saddle point at α(t)=0.53; α(t) then increases to the free-diffusion limit α(t)=1 at the longest times studied.

Adeyemi et al. [25] simulated bidisperse blend melts with the Kremer-Grest model. The melts contained a long (350 bead) polymer and a shorter polymer, the shorter chains having 25, 50, or 100 beads. Comparison was made with monodisperse melts of chains having each of the four chain lengths. The blends were either 0.7 or 0.3 by volume fraction of the longer chains, and therefore 0.3 or 0.7 by volume fraction of the shorter chains. The molecular weights follow those in Wang and Larson [26], except that here neither polymer species in the blend is dilute. Comparison was made with simulations of the four polymers in single component systems. The authors determined the center-of-mass displacement function $g_{3}\left(t\right)$ and, for the monodisperse chains, the single-bead displacement function $g_{1}\left(t\right)$. To avoid chain-end effects, the reported $g_{1}\left(t\right)$ was taken as the motion of the central bead of each chain, not the motions of all the beads of each chain. They further examined, not considered here, the dynamics of the Rouse modes and stress relaxation following a step shear strain.

By force-fitting the observed $g_{1}\left(t\right)$ for the monodisperse systems to tube-reptation predictions for $\tau_{o}$ and $\tau_{e}$, Adeyemi et al., estimated for a monodisperse system that the number $N_{e}$ of beads between adjoining entanglements was $N_{e}\approx 33$. This result agrees with Kremer and Grest [39]. It follows that Adeyemi et al.’s three shorter polymers were unentangled or barely entangled (Z≈3), while their long polymer averaged Z≈10 entanglements.

**Figure 12:**
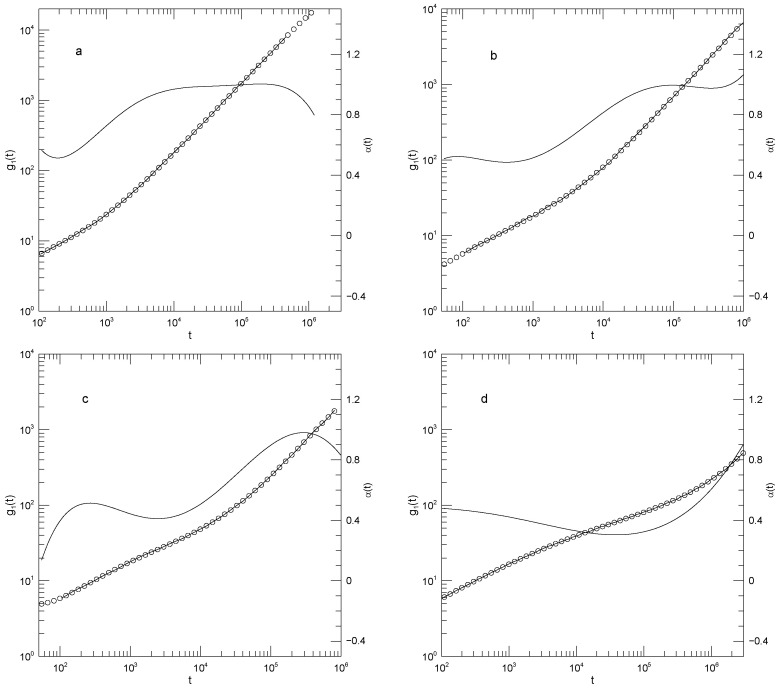
Mean-square central bead displacements $g_{1}\left(t\right)$ (thick lines) of melts of monodisperse Kremer-Grest bead-spring chains, based on simulations of Adeyemi et al. [25], together with fits to eighth-order polynomials (circles) and the corresponding first logarithmic derivatives α(t) (thin lines). Chains contained (**a**) 25, (**b**) 50, (**c**) 100, or (**d**) 350 beads. Note the long-time behavior seen in Figure 12a. In that Figure, the fitted curve (open circles) has been extrapolated beyond a time $t\approx 4\times 10^{5}$, this being the largest time reported in the simulated data. Before the extrapolated region is reached, there is an extended region in which α(t) is approximately constant, corresponding to power-law behavior of g(t). At times $t>4\times 10^{5}$, the slope of the extrapolated fitted curve decreases slightly with increasing *t*, and correspondingly at these larger times α(t) decreases with increasing *t*, as seen in the Figure. The change in slope at these larger times is only barely visible to the naked eye, even with the help of a straight-edge, showing the sensitivity of our data analysis technique.

Figure 12 presents Adeyemi et al.’s measurements of $g_{1}\left(t\right)$ for their four polymers in monodisperse melts. For all polymers, α(t) is close to 0.5 at early times, and at long times reaches α(t)≈1.0. For the 50-bead polymer, these values of α(t) correspond to manifest power-law regimes. With increasing chain length, a minimum in α(t) appears at intermediate times, the minimum increasing in depth and width with increasing chain length. The minimum is scarcely visible for the 50-bead polymer, bottoms out at α(t)=0.41 for the hundred bead polymer, and declines to a broad α(t)≈0.3 for the 350-bead polymer. These are all minima, not places where α(t) is a local constant, as would be seen if power-law behavior were present at intermediate times. The transition regions between the $t^{0.5}$ and $t^{1}$ behaviors are quite wide.

**Figure 13:**
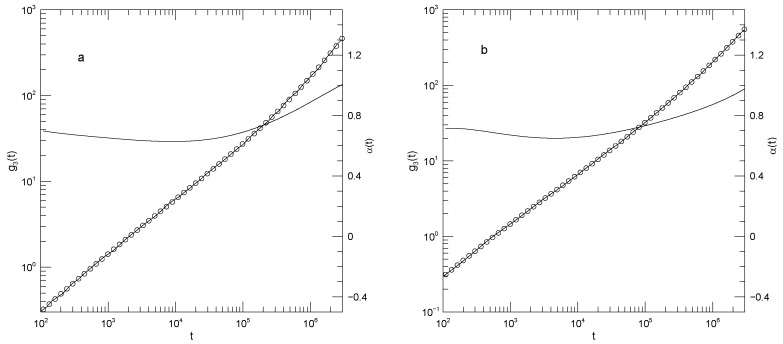

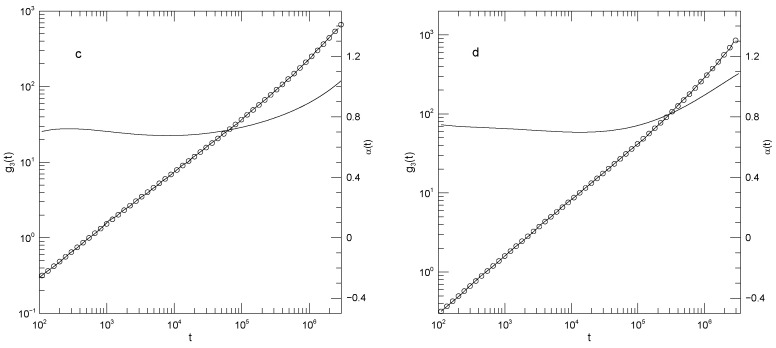
Mean-square center-of-mass displacements $g_{3}\left(t\right)$ (thick lines) of 350-bead chains in melt blends of Kremer-Grest bead-spring chains, based on simulations of Adeyemi et al. [25], together with fits to eighth-order polynomials (circles), and the corresponding first logarithmic derivatives α(t) (thin lines). Figure part (**a**) refers to a pure 350-bead melt. Figures (**b**–**d**) refer to blends with 0.7 volume fraction of the 350-bead polymer and 0.3 volume fraction of the (**b**) 100-bead, (**c**) 50-bead, and (**d**) 25-bead polymer.

Figure 13 shows the center-of-mass displacements $g_{3}\left(t\right)$ for the pure long-chain polymer and for the long-chain polymer mildly diluted ($\Phi_{L}=0.7,\Phi_{S}=0.3$) by each of the three shorter-chain polymers. In summary, at shorter times ($t<10^{4}$) the mean-square center-of-mass displacements approach power-law behavior with exponents near α≈0.7, the approach being closest with dilution by the lightest polymer. At longer times, out to the longest times observed, α(t) increases progressively with increasing time, with no sign that a long-time $t^{1}$ regime has been reached. It is not obvious that α(t) has a maximum at the longest time reported. Near its minimum, α(t) for each system is clearly concave upward; correspondingly, near this point $g_{3}\left(t\right)$ has an inflection point, not power-law behavior.

Diluting the long-chain polymer with modest amounts of the shorter chains, as seen in Figure 13b–d, has only a modest effect on $g_{3}\left(t\right)$. α(t) at the shortest times studied gradually increases from 0.7 (for dilution of the 350-bead polymer with the 100-bead polymer) to 0.77 (for dilution with the 25-bead polymer). At its minimum, α(t) from $g_{3}\left(t\right)$ increases from 0.63 in pure long-chain polymer to 0.70 for dilution of the 350-bead polymer by the shortest, 25-bead, polymer.

**Figure 14:**
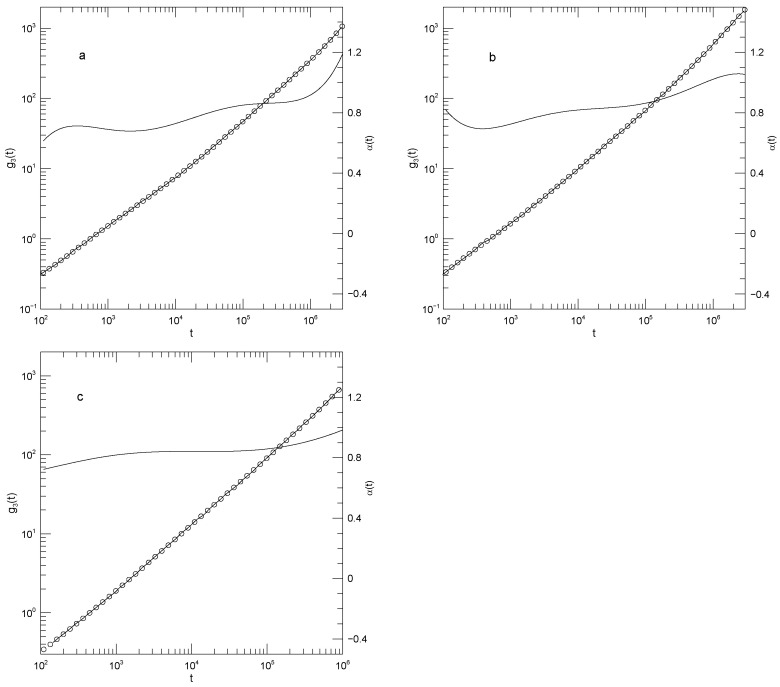
Mean-square center-of-mass displacements $g_{3}\left(t\right)$ (thick lines) of 350-bead chains in blends of Kremer-Grest bead-spring chains, based on simulations of Adeyemi et al. [25], together with fits to eighth-order polynomials (circles), and the corresponding first logarithmic derivatives α(t) (thin lines). The figures (**a**–**c**) refer to blends with 0.3 volume fraction of the 350-bead polymer and 0.7 volume fraction of the (**a**) 100-bead, (**b**) 50-bead, and (**c**) 25-bead polymer.

Figure 14 shows $g_{3}\left(t\right)$ for the 350-bead polymer that has been heavily diluted ($\Phi_{L}=0.3$, $\Phi_{S}=0.7$) with shorter chains. At this dilution, $g_{3}\left(t\right)$ is nearly featureless in all three diluents. When mixed with the 100-bead or 50-bead diluents, α(t) increases from ≈0.7 to ≈0.9–1.0 over the observed range of times. When combined with the 25-bead polymer, over an extended range $g_{3}\left(t\right)$ does show power-law behavior, with exponent α(t)≈ 0.84–0.85.

Finally, as part of our demonstration in our previous papers [20,21] that our method can identify power-law regions when they are present, we examined results of Brodeck, et al. [15] and Moreno and Colmenero [27]. These authors among other properties examined $g_{1}\left(t\right)$ of their chains. Their results indicate that $g_{1}\left(t\right)$ has at most a single power-law regime, not the multiple power-law regimes predicted in some tube models.

## 4. Discussion

We examined the time dependences of $g_{1}\left(t\right)$ and $g_{3}\left(t\right)$ in a series of monodisperse and bidisperse systems. Comparison was made with tube-reptation-scaling model predictions $g_{1}\left(t\right)∼t^{\alpha }$ for the time dependence of the mean-square displacements. For completeness we also reported the less-interesting $g_{2}\left(t\right)$, which increases and sometimes reaches its long-time behavior, in which $g_{2}\left(t\right)$ is a constant. We searched for power-law behaviors. Long-time diffusive behavior (α(t)=1) was sometimes seen. At earlier times, at most one power-law regime was observed.

We turn first to the single-bead motions described by $g_{1}\left(t\right)$. In general, at short times α(t) is large, corresponding to a rapid initial increase of $g_{1}\left(t\right)$. α(t) then decreases to a local minimum, corresponding to an inflection point in $g_{1}\left(t\right)$. Particularly for the shorter chains and shorter matrix chains, following the inflection point α(t) has a single plateau corresponding to a single power-law regime in $g_{1}\left(t\right)$. After the plateau, $g_{1}\left(t\right)$ increases again, reaching at the longest times studied diffusive ($t^{1}$) behavior.

In more detail, the depth of the local minimum depends on the system being observed, but was 0.2 or 0.46 for the two polymers in Sacristan et al.’s blend [23]. From Kopf, et al.’s results [24], the time at which the minimum is seen increases with increasing polymer molecular weight, from ∼$10^{2}$ for their 20-bead blend to ∼$10^{3}$ for their 50-bead blend and ∼$10^{4}$ for their 150-bead polymers. Kopf et al., created a blend by changing the mass of the beads of some of their polymer chains. Changing the mass had only a modest effect on $g_{1}\left(t\right)$. Wang and Larson [26] observed 350-bead chains in shorter-chain matrices. They observed slightly more rapid motion at earlier times, an extended plateau or broad minimum in α(t) at intermediate times, and with the longer matrix polymers an increase in α(t) at large times. The plateau corresponds to a single power-law or near-power-law regime, with exponent falling from 0.46 to 0.30 with increasing blend matrix molecular weight, and finally to α(t)≈0.27 for the plateau in the pure melt. Adeyemi et al. [25] extended their measurements to a sufficient time that α(t)≈1 diffusive behavior was clearly seen at long times. At short times, their α(t) had structure, including for different polymer lengths an early increase followed by a saddle point, the slope at the saddle point being in the range 0.3–0.4. As discussed in our previous papers [20,21] Brodeck et al. [15] and Moreno and Colmenero [27] consistently found at most a single power-law regime.

We may also consider the center-of-mass motions described by $g_{3}\left(t\right)$. For very short chains, Sacristan, et al’s results, Figure 2 find that α(t) is large at early times, decreases to a local minimum, and then increases into a plateau, revealing a single local power-law regime with exponent α(t)≈2/3. Kopf et al. [24] report $g_{3}\left(t\right)$ over two-and-a-half orders of magnitude in time, during which $g_{3}\left(t\right)$ has a nearly featureless increase. For their 20-bead polymers, a weak saddle point is seen at early times. For their N=30 chains in their blend, $g_{3}\left(t\right)$ for either blend component is very gently curving, corresponding to an exponent in the range 0.9–1.0. Their results show near-power-law behavior with a single exponent. Wang and Larson [26] examine 350-bead polymers in melts of 25, 50, 80, or 160-bead polymers, finding that in the 160-bead polymer melt α(t) has a weak saddlepoint. As we progress to shorter and shorter matrix polymers, the curvature in the plot of $g_{3}\left(t\right)$ fades. In the 25-bead melt, $g_{3}\left(t\right)$ for $t>10^{3}$ is very close to a power law with α(t)≈0.9. Finally, Adeyemi et al. [25] examined $g_{3}\left(t\right)$ for 350-bead chains mixed with shorter chains in 70:30 and 30:70 mixtures. When the long chain was the major component, at times $t\leq 10^{5}$, we find $g_{3}\left(t\right)$ is close to a power law with exponent ≈3/4. At larger times the slope α(t) smoothly and progressively increases to α(t)≈1. In the 30:70 blend, the log-log plots of Figure 14 show that $\log(g_{3}\left(t\right))$ is weakly concave upward. α(t) increases by a tenth or two in the different blends over most of the times examined. The shorter the length of the short polymer, the closer a region of $g_{3}\left(t\right)$ approaches to power-law behavior. In the blend with the 25-bead polymer, a single power-law region is seen, with α(t)≈5/6.

From our fits to log-log plots, we see that at intermediate times $g_{1}\left(t\right)$, $g_{2}\left(t\right)$, and $g_{3}\left(t\right)$ have at most a single power-law regime, the time dependence of their logarithmic derivative α(t) at all other times being described by a curve of continuously changing slope.

Several authors have advanced theoretical treatments of binary blends of a polymer [40,41,42,43]. Note in particular results of Doi et al. [18] and Viovy et al. [19]. These models propose that the motions of a high-molecular-weight polymer may be understood by imagining that the polymer’s motions are restricted by its neighbors. Each polymer molecule is free to move more-or-less parallel to its own contour, but is seriously inhibited from moving perpendicular to its chain contour. However, there are processes (‘tube dilation’, ‘tube renewal’, ‘constraint release’) that gradually relax the polymer’s constraints against motion transverse to its original contour. The relative importance of these processes can, these authors say, be identified by considering the motions of a given polymer as the molecular weight of the surrounding polymers—the matrix polymers—is changed. Of particular interest to these authors were systems in which the polymer of interest was of higher molecular weight than the surrounding matrix polymers. With respect to our results, the important result of these authors (Doi et al. [18] their Figures 5 and 6; Viovy et al. [19] their Figures 3–5) is the prediction that the mean-square displacements follow a series of power laws, the power-law exponents depending on the time over which the mean-square displacements are determined, and the times at which the exponent changes from one value to the next being a central part of their theoretical analysis.

Our findings here are quite different from these predictions that the g(t) are each described as a series of power laws. We find for a polymer chain in a blend melt that the mean-square displacement has at most one power-law region. Outside of that region, we find that log-log plots of mean-square displacement against time follow smooth curve whose slope varies continually with time. Because we find that the mean-square displacements do not have the qualitative forms proposed by Doi et al., or Viovy et al., their models are apparently incorrect. There then does not appear to be a rationale for making a detailed numerical comparison with their quantitative predictions.

It could be proposed that the transition regions between the hypothesized power-law regimes are quite wide, so that the power-law regimes have been more-or-less completely swallowed up by the transitions between them. However, in this case theories treating the power-law regions would be describing a very small part of the time dependence. An important theoretical objective would instead be to predict the smooth curves of the transition regions, but research on the forms of the g(t) appear to have focused elsewhere.

## Data Availability

Data are contained within the article.

## Supplementary Materials

The following supporting information can be downloaded at: https://www.mdpi.com/article/10.3390/polym17233140/s1: Figures S1a to S14c of the Supplemental Material reproduce at full size Figure 1, Figure 2, Figure 3, Figure 4, Figure 5, Figure 6, Figure 7, Figure 8, Figure 9, Figure 10, Figure 11, Figure 12, Figure 13 and Figure 14 of the text, thus permitting detailed examination of the quality of our fits.
