# Supplementary material for: Mean-Square Displacements of Polymers in Simulated Blend Melts
Source: Polymers (Basel). 2025 Nov 26;17(23):3140. doi: 10.3390/polym17233140 (PMC12694126; doi:10.3390/polym17233140)
Supplement: Supplementary file 1 [file polymers-17-03140-s001.zip › polymers-3988443-supplementary.pdf]

Supplemental Material

The Supplemental Material reproduces at full scale the text figures, so that the quality of the fits may more easily be appreciated. Figure and Citation numbers are the same as in the main text, so that, e.g., Figure S-1a of the Supplemental Material is the same as Figure 1a of the text.

Figure S-1a) Mean-square center-of-mass displacement $g_1(t)$ (thick line), our fit of $g_1(t)$ to an eighth-order polynomial (circles), and the corresponding first logarithmic derivative $\alpha(t)$ (thin line). The Figure shows (a) polyethylene oxide chains in a polyethylene oxide-polymethylmethacrylate blend, based on simulations by Sacristan, et al. [23].

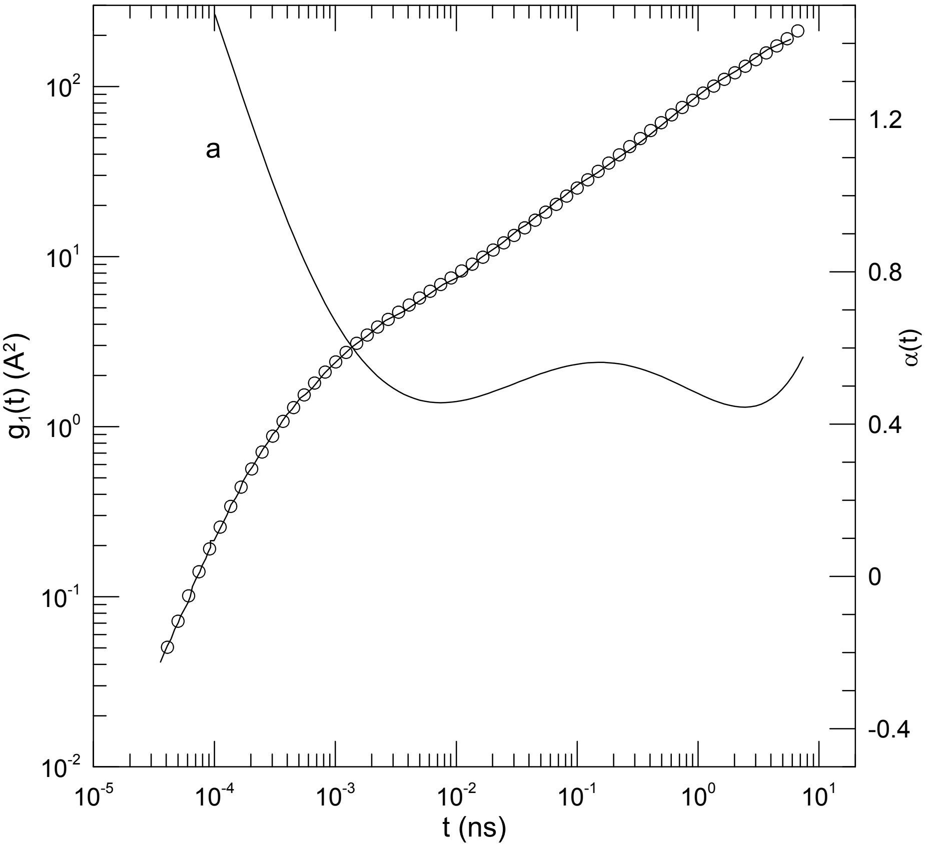

Figure S-1b) Mean-square center-of-mass displacement $g_1(t)$ (thick line), our fit of $g_1(t)$ to an eighth-order polynomial (circles), and the corresponding first logarithmic derivative $\alpha(t)$ (thin line). The Figure shows (b) polymethylmethacrylate chains in a polyethylene oxide-polymethylmethacrylate blend, based on simulations by Sacristan, et al. [23].

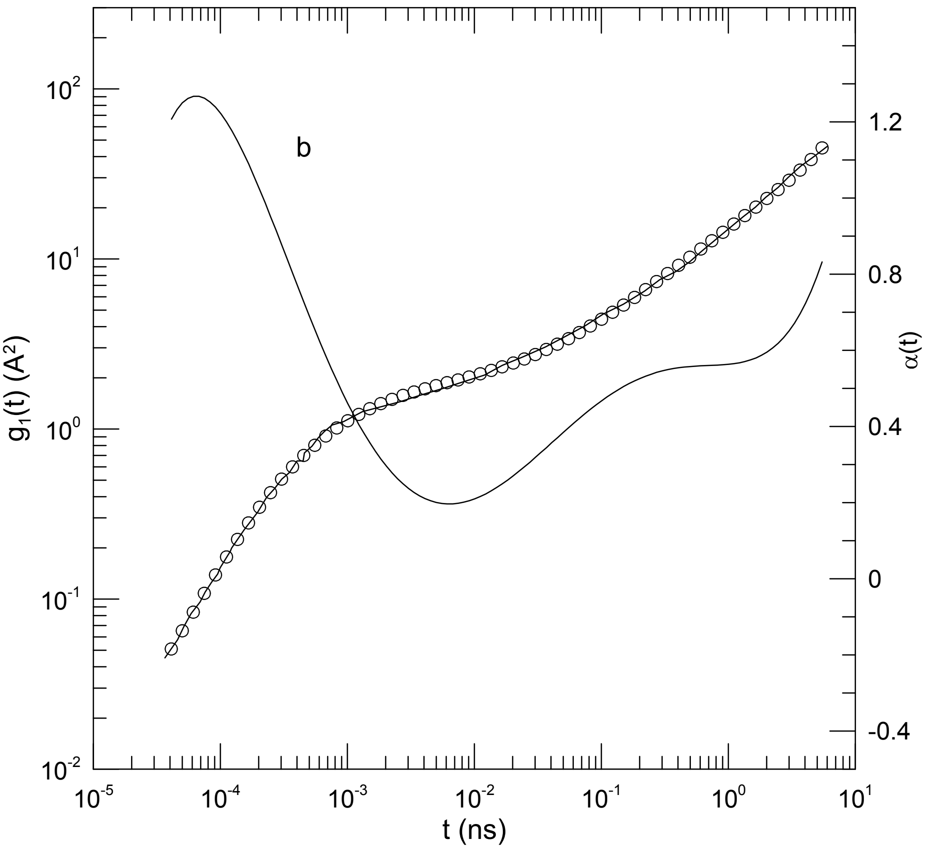

Figure S-1c) Mean-square center-of-mass displacement $g_1(t)$ (thick line), our fit of $g_1(t)$ to an eighth-order polynomial (circles), and the corresponding first logarithmic derivative $\alpha(t)$ (thin line). The Figure shows (c) the polyethylene oxide segment of a polyethylene oxide-polymethylmethacrylate diblock copolymer, based on simulations by Sacristan, et al. [23].

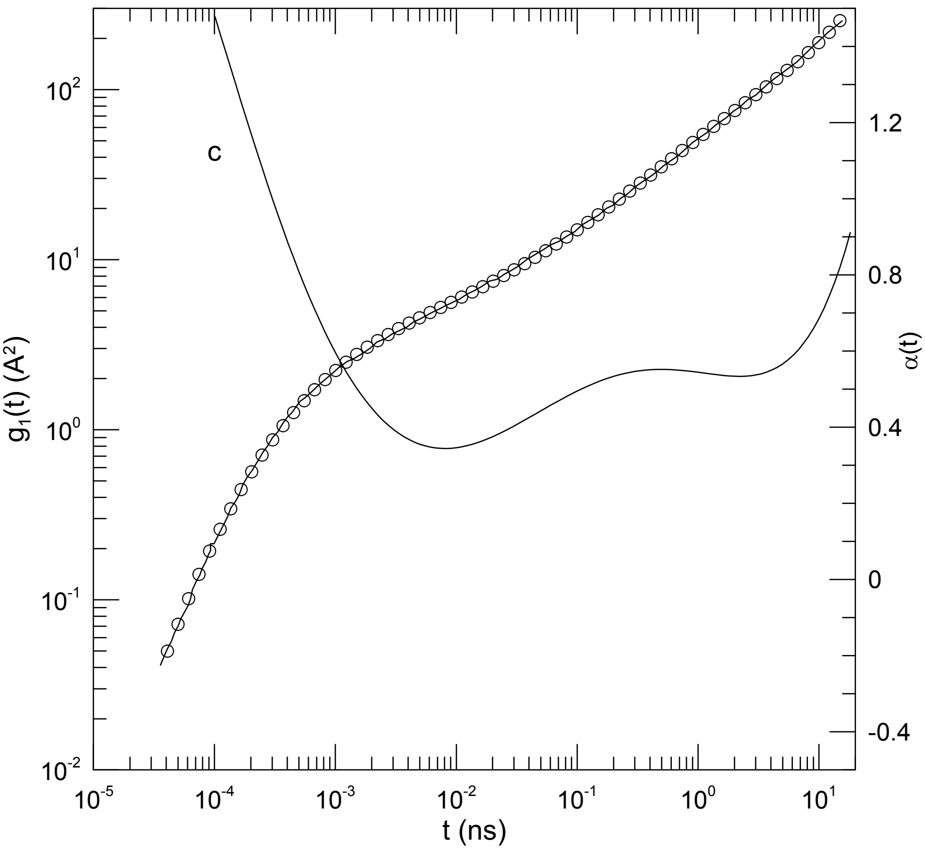

Figure S-1d) Mean-square center-of-mass displacement $g_1(t)$ (thick line), our fits of $g_1(t)$ to an eighth-order polynomial (circles), and the corresponding first logarithmic derivative $\alpha(t)$ (thin lines). The Figure shows (d) the polymethylmethacrylate segment of a polyethylene oxide-polymethylmethacrylate diblock copolymer, based on simulations by Sacristan, et al. [23].

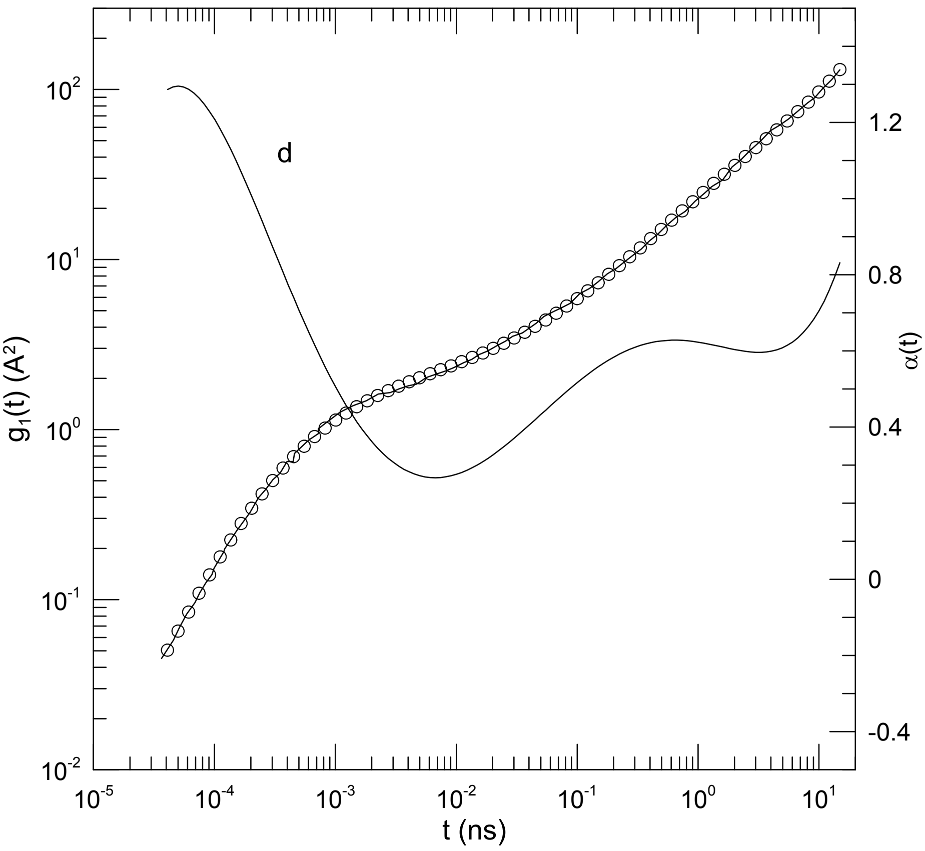

Figure S-2a) Mean-square center-of-mass displacement $g_3(t)$ (thick line), our fit of $g_3(t)$ to an eighth-order polynomials (circles), and the corresponding first logarithmic derivative $\alpha(t)$ (thin line). The figure shows (a) polyethylene oxide chains in a polyethylene oxide-polymethylmethacrylate blend, based on simulations of Sacristan, et al. [23].

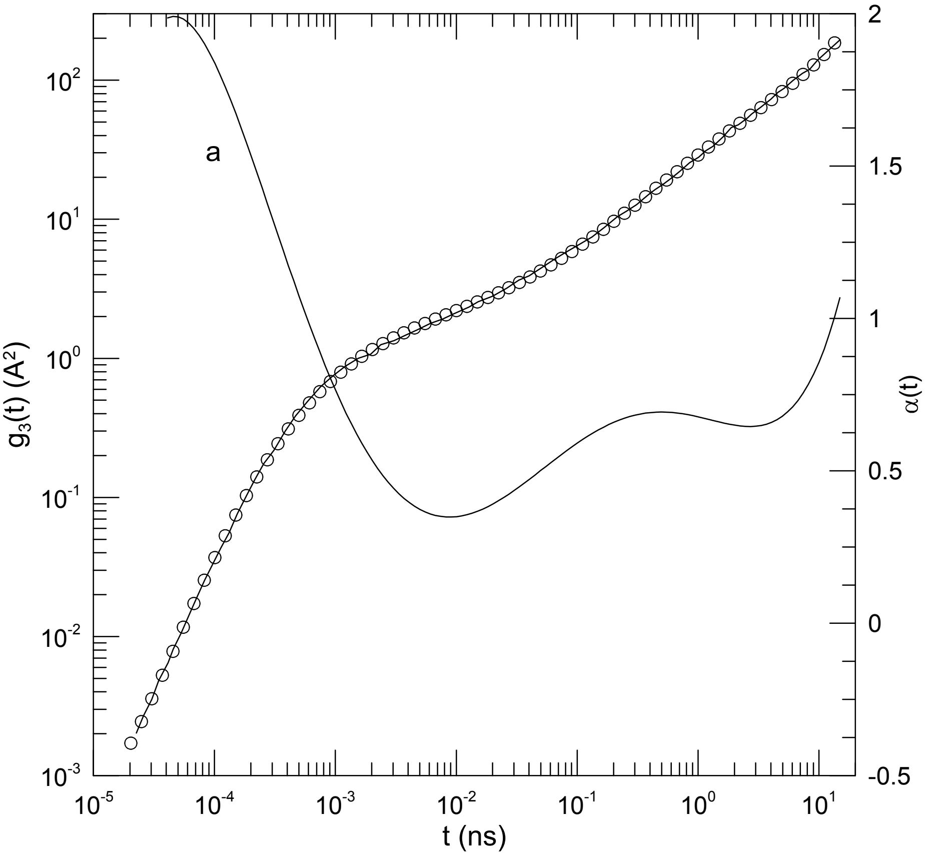

Figure S-2b) Mean-square center-of-mass displacement $g_3(t)$ (thick line), our fit of $g_3(t)$ to an eighth-order polynomial (circles), and the corresponding first logarithmic derivative $\alpha(t)$ (thin line). The Figure shows (b) a polyethylene oxide:polymethylmethacrylate block copolymer, based on simulations of Sacristan, et al. [23].

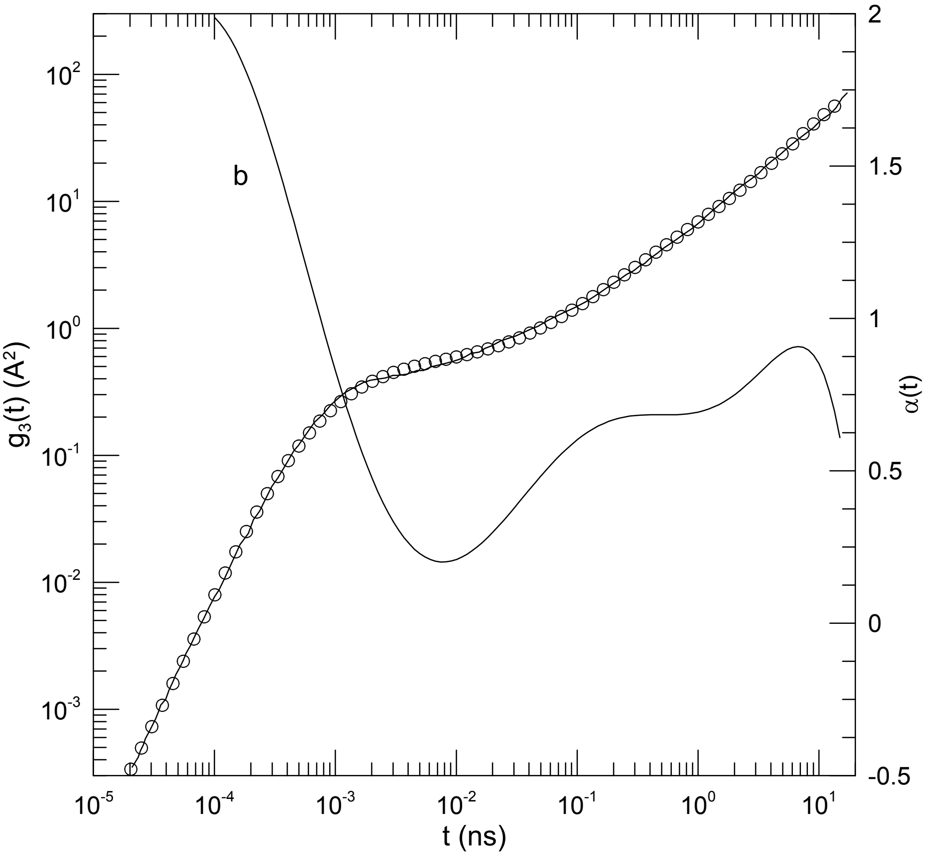

Figure S-3a) Mean-square central bead displacement $g_1(t)$ (thick line) of melts of Kremer-Grest bead-spring chains, based on simulations of Kopf, et al. [24], together with a fit to an eighth-order polynomial (circles), and the corresponding first logarithmic derivative $\alpha(t)$ (thin line). Chains contained (a) 20 beads.

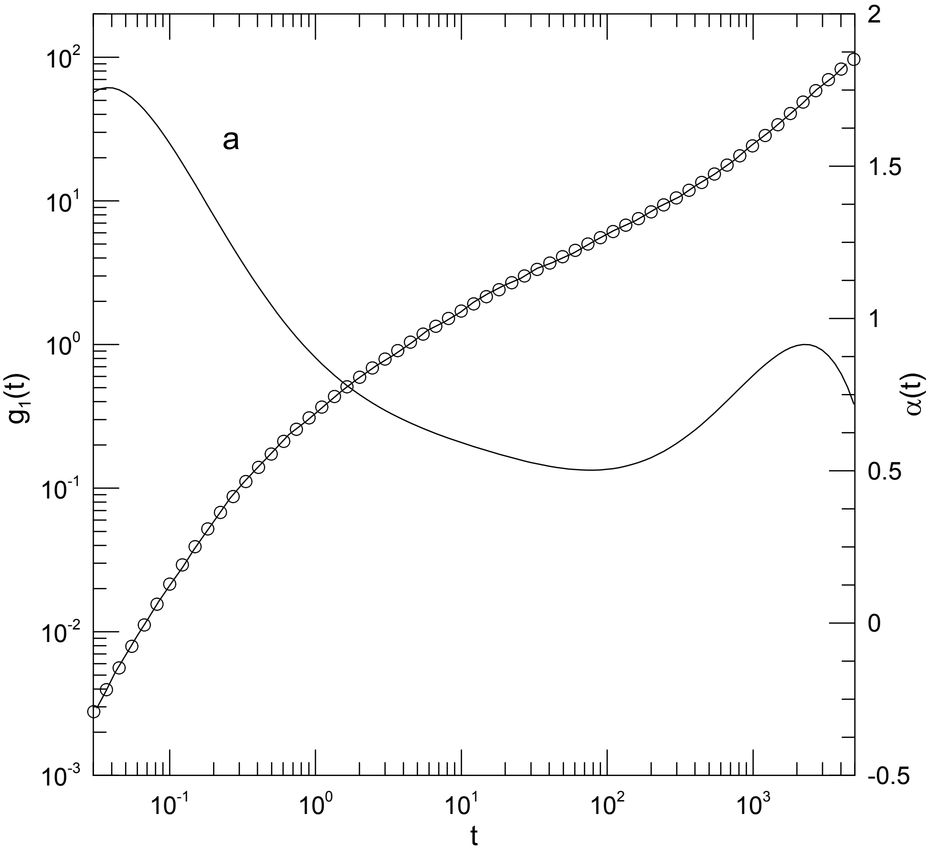

Figure S-3b) Mean-square central bead displacement $g_1(t)$ (thick line) of melts of Kremer-Grest bead-spring chains, based on simulations of Kopf, et al. [24], together with a fit to an eighth-order polynomial (circles), and the corresponding first logarithmic derivative $\alpha(t)$ (thin lins). Chains contained (b) 30 beads.

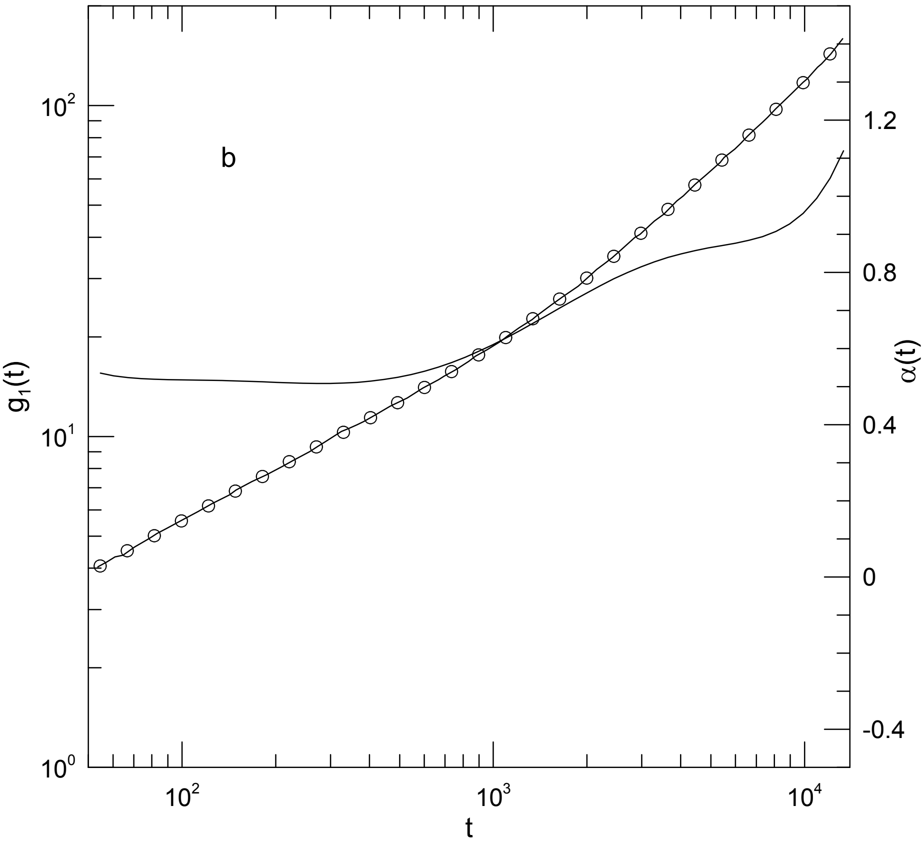

Figure S-3c) Mean-square central bead displacement $g_1(t)$ (thick line) of melts of Kremer-Grest bead-spring chains, based on simulations of Kopf, et al. [24], together with a fit to an eighth-order polynomial (circles), and the corresponding first logarithmic derivative $\alpha(t)$ (thin line). Chains contained (c) 50 beads.

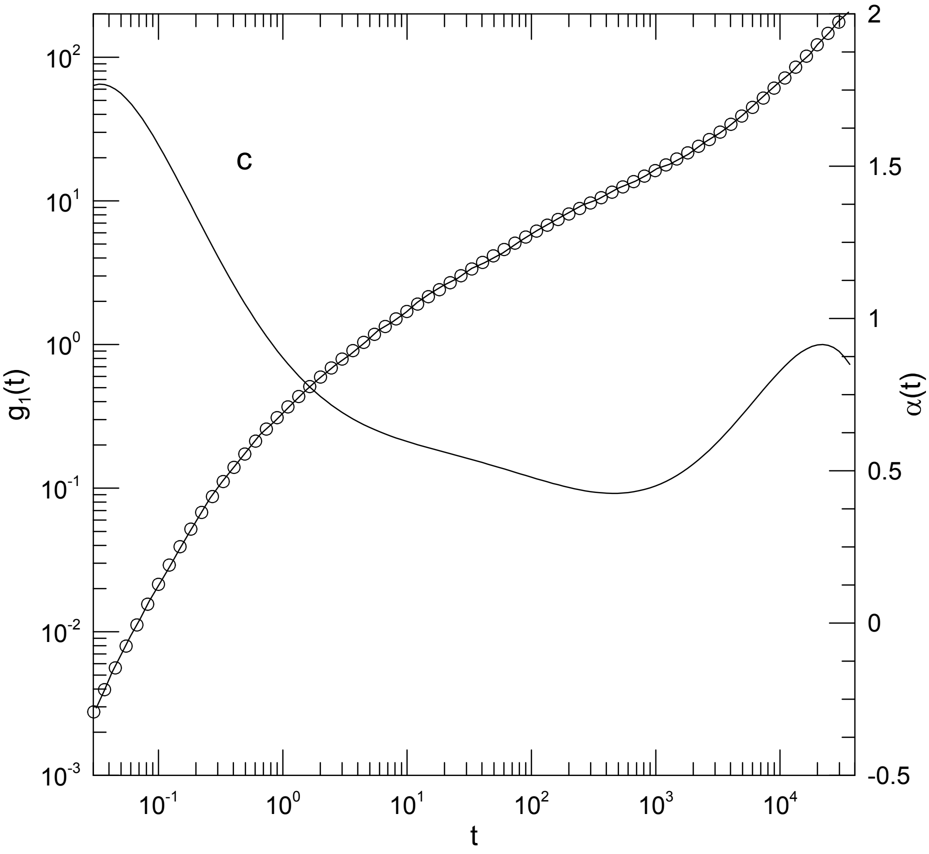

Figure S-3d) Mean-square central bead displacement $g_1(t)$ (thick line) of melts of Kremer-Grest bead-spring chains, based on simulations of Kopf, et al. [24], together with a fit to an eighth-order polynomial (circles), and the corresponding first logarithmic derivative $\alpha(t)$ (thin line). Chains contained (d) 150 beads.

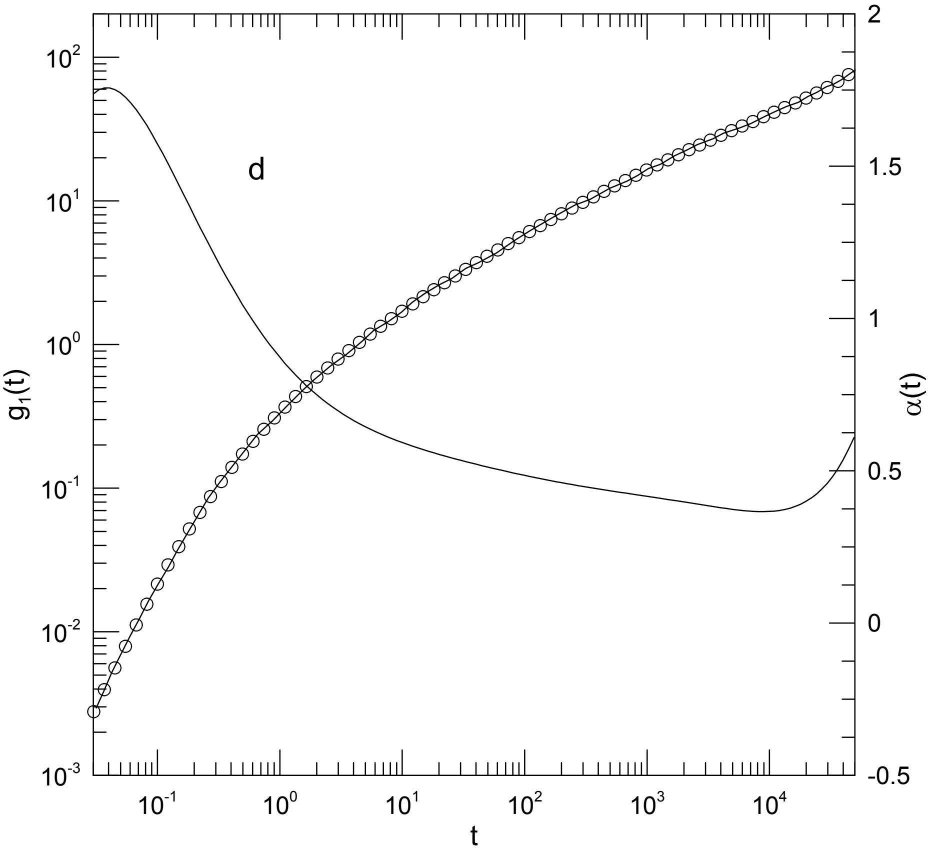

Figure S-4a) Mean-square center-of-mass displacement $g_3(t)$ (thick line) of melts of Kremer-Grest bead-spring chains, based on simulations of Kopf, et al. [24], together with a fit to an eighth-order polynomial (circles), and the corresponding first logarithmic derivative $\alpha(t)$ (thin line). Chains contained (a) 20 beads.

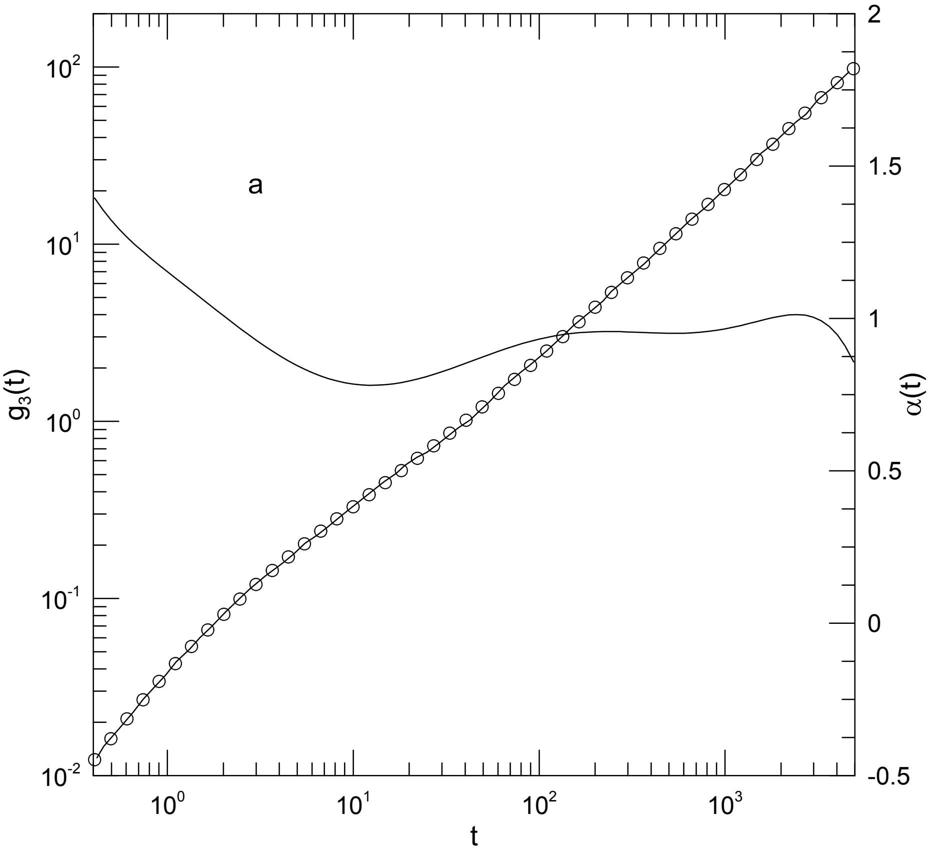

Figure S-4b) Mean-square center-of-mass displacement $g_3(t)$ (thick line) of melts of Kremer-Grest bead-spring chains, based on simulations of Kopf, et al. [24], together with a fit to an eighth-order polynomials (circles), and the corresponding first logarithmic derivative $\alpha(t)$ (thin line). Chains contained (b) 30 beads.

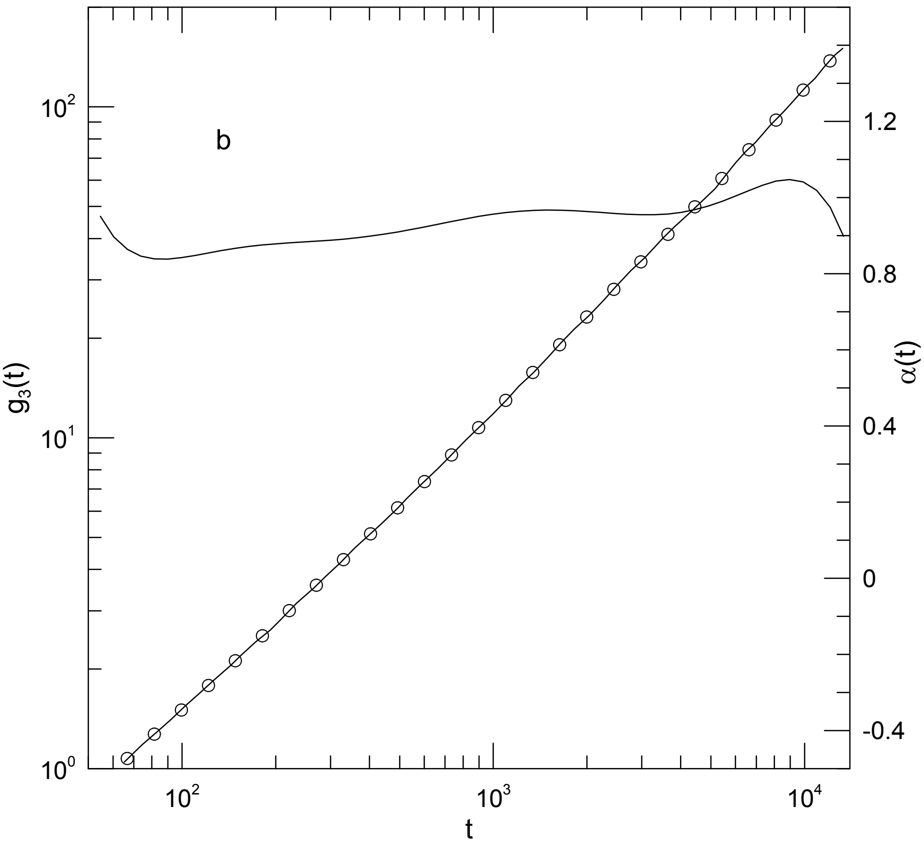

Figure S-4c) Mean-square center-of-mass displacement $g_3(t)$ (thick line) of melts of Kremer-Grest bead-spring chains, based on simulations of Kopf, et al. [24], together with a fit to an eighth-order polynomial (circles), and the corresponding first logarithmic derivative $\alpha(t)$ (thin line). Chains contained (c) 50 beads.

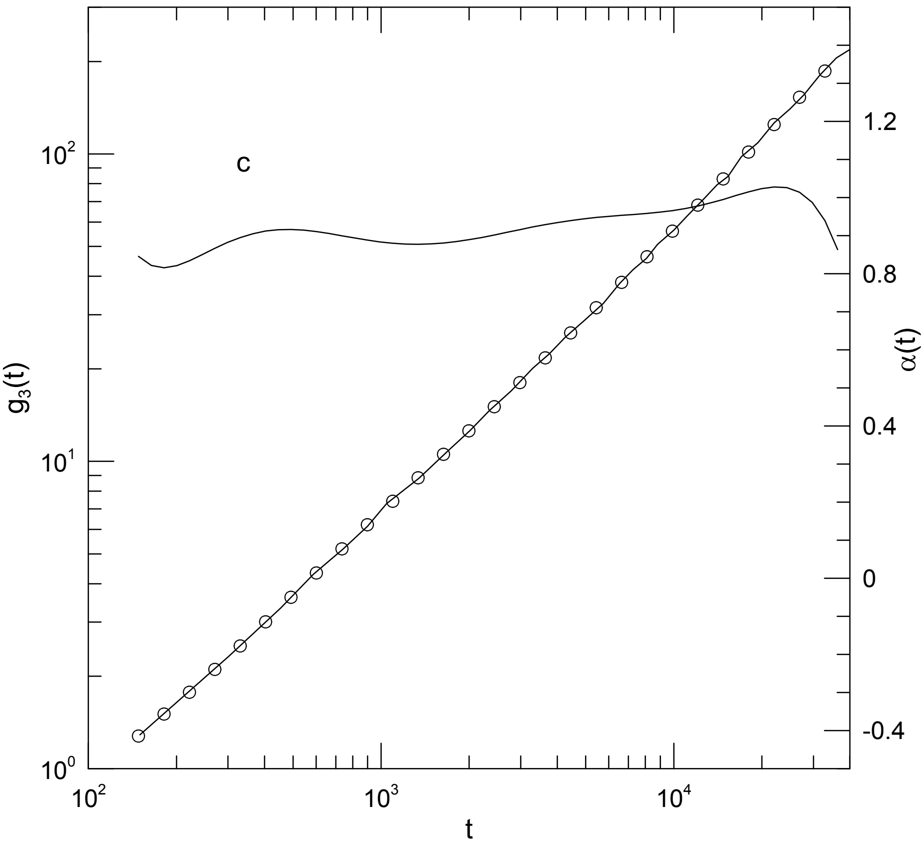

Figure S-4d) Mean-square center-of-mass displacement $g_3(t)$ (thick line) of melts of Kremer-Grest bead-spring chains, based on simulations of Kopf, et al. [24], together with a fit to an eighth-order polynomial (circles), and the corresponding first logarithmic derivatives $\alpha(t)$ (thin line). Chains contained (d) 150 beads.

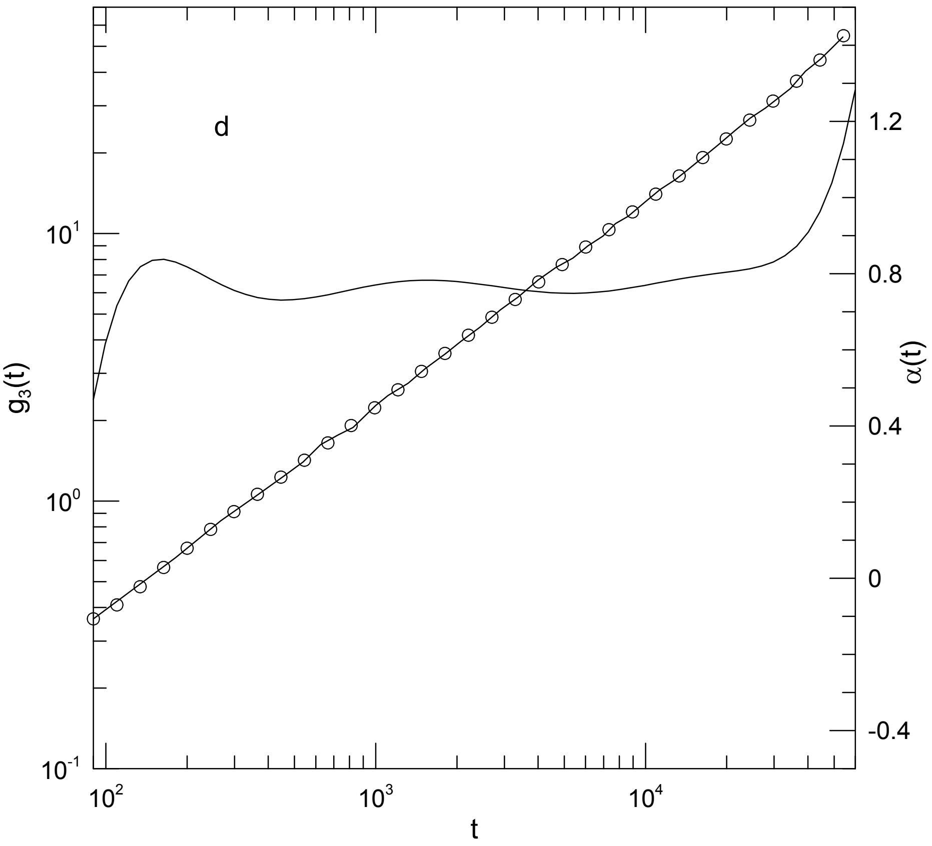

Figure S-5a) Mean-square displacement $g_1(t)$ (thick line) of the light chains in a 50:50 light-heavy polymer blend of Kremer-Grest bead-spring chains, based on simulations of Kopf, et al. [24], together with a fit to an eighth-order polynomial (circles), and the corresponding first logarithmic derivative $\alpha(t)$ (thin line). All chains had $N = 30$; the light chains had $m = 1$; the heavy chains had $m = 4$.

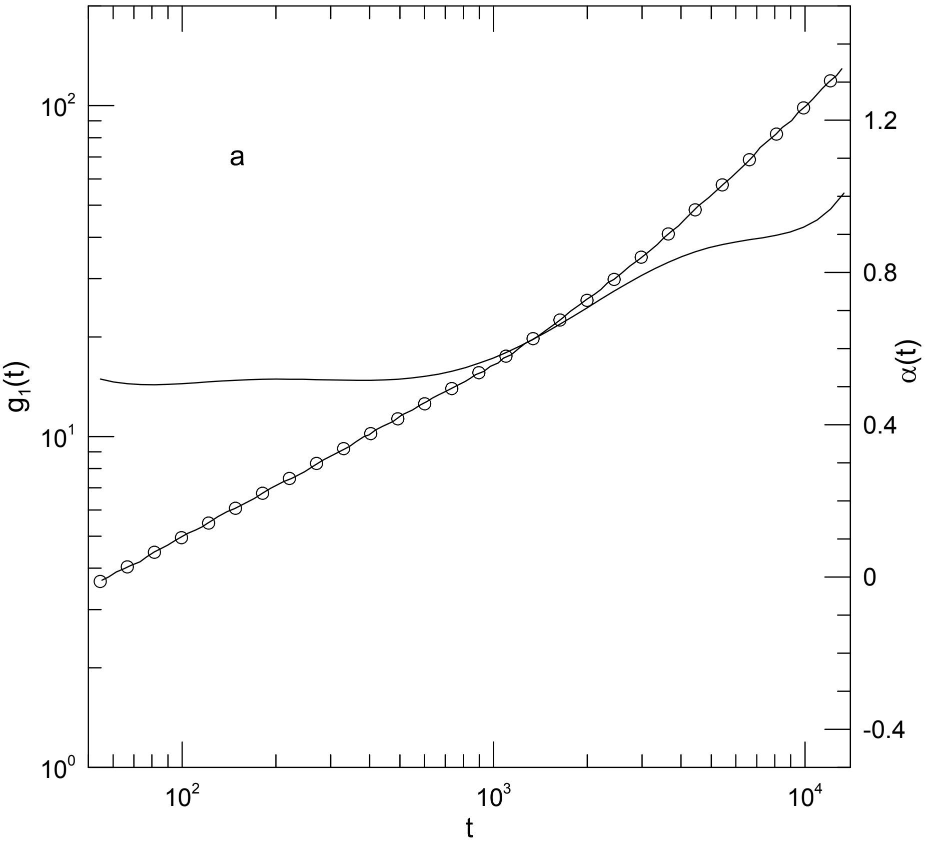

Figure S-5b) Mean-square displacement $g_3(t)$ (thick line) of the light chains in a 50:50 light-heavy polymer blend of Kremer-Grest bead-spring chains, based on simulations of Kopf, et al. [24], together with a fit to an eighth-order polynomial (circles), and the corresponding first logarithmic derivative $\alpha(t)$ (thin line). All chains had $N = 30$; the light chains had $m = 1$; the heavy chains had $m = 4$.

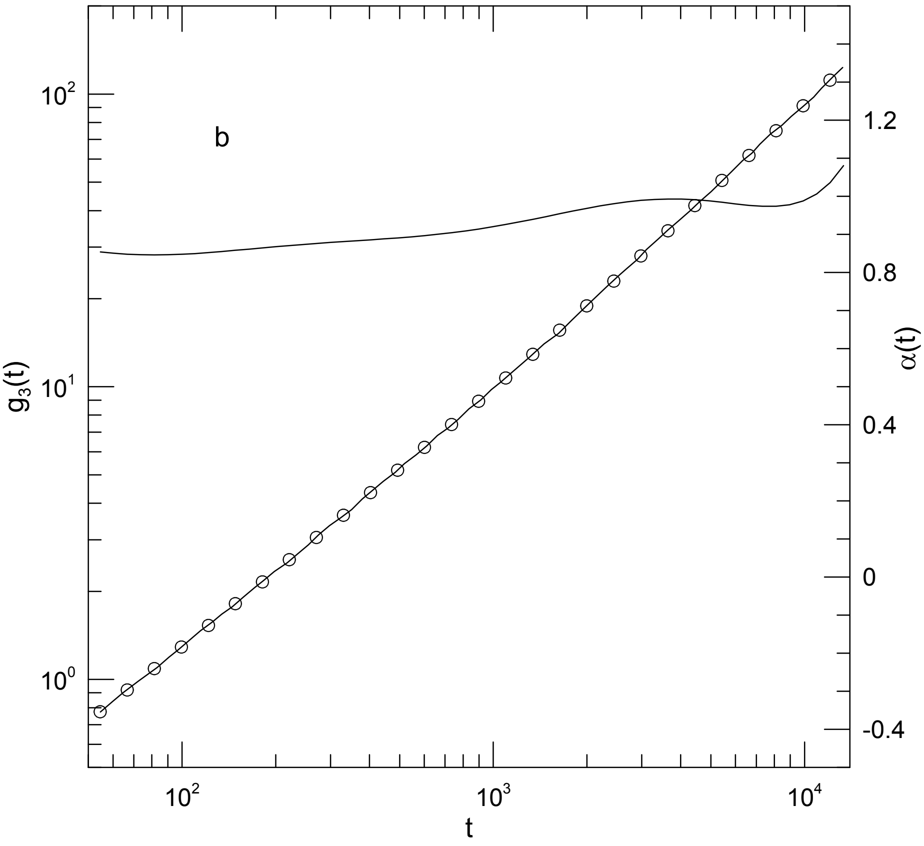

Figure S-5c) Mean-square displacement $g_1(t)$ (thick line) of the light chains in a 50:50 light-heavy polymer blend of Kremer-Grest bead-spring chains, based on simulations of Kopf, et al. [24], together with a fit to an eighth-order polynomial (circles), and the corresponding first logarithmic derivative $\alpha(t)$ (thin line). All chains had $N = 30$; the light chains had $m = 1$; the heavy chains had $m = 100$.

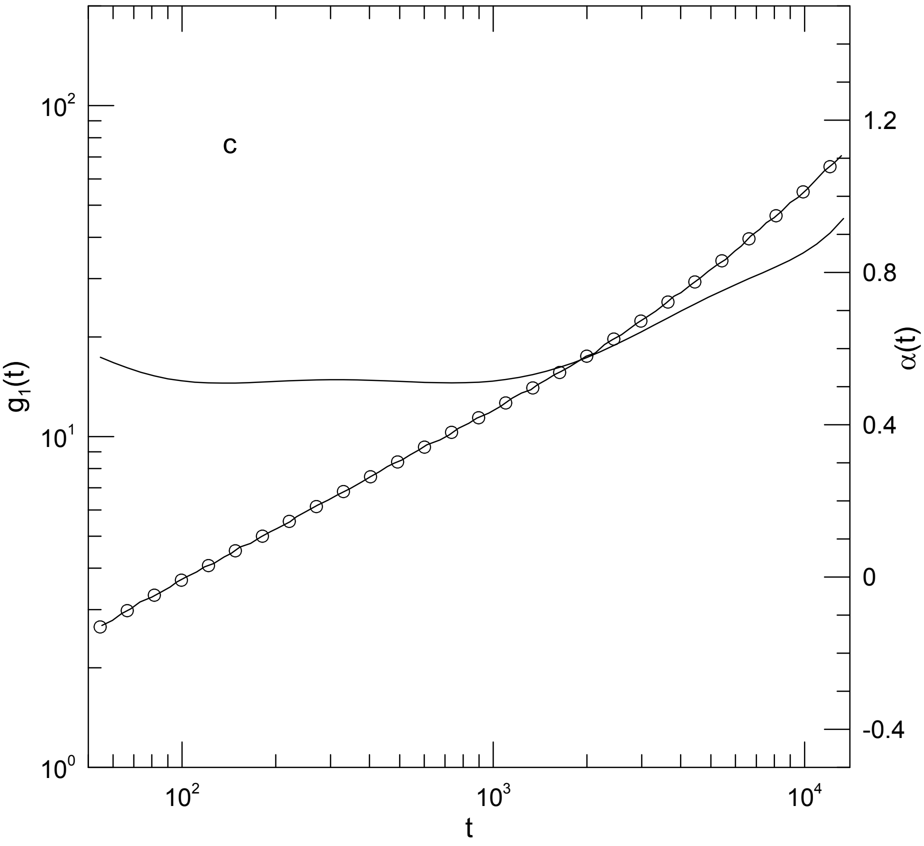

Figure S-5d) Mean-square displacement $g_3(t)$ (thick line) of the light chains in a 50:50 light-heavy polymer blend of Kremer-Grest bead-spring chains, based on simulations of Kopf, et al. [24], together with a fits to an eighth-order polynomial (circles), and the corresponding first logarithmic derivative $\alpha(t)$ (thin line). All chains had $N = 30$; the light chains had $m = 1$; the heavy chains had $m = 100$.

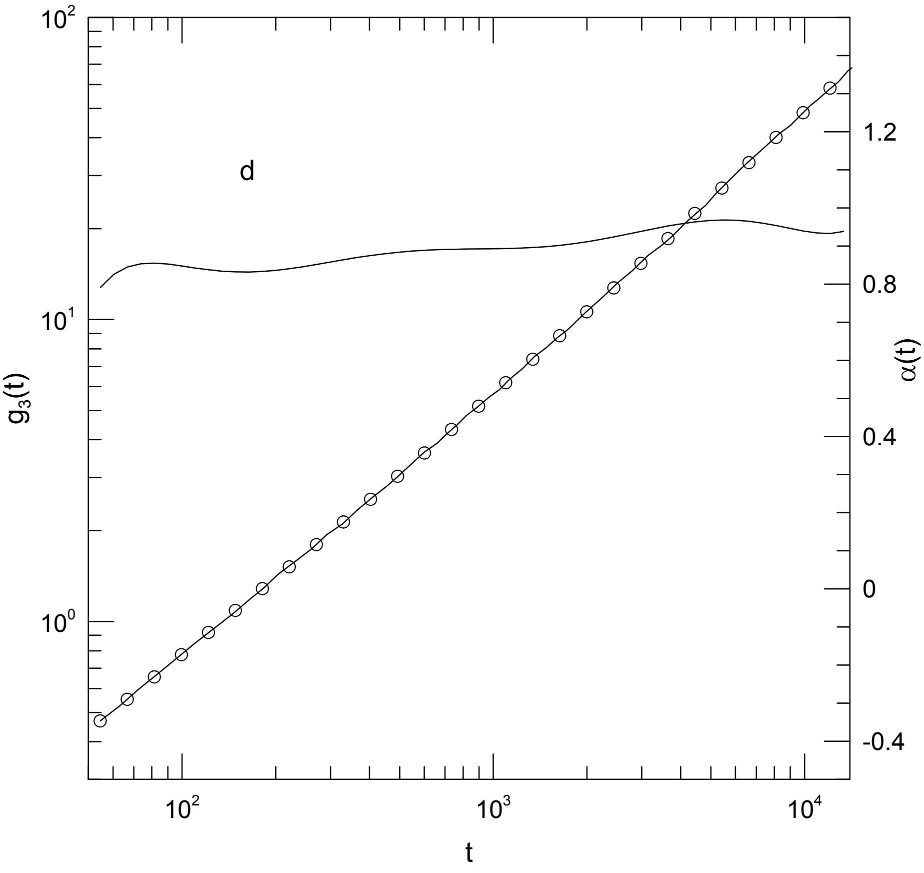

Figure S-6a) Mean-square displacement $g_1(t)$ (thick line) of the heavy chains in a 50:50 light-heavy polymer blend of Kremer-Grest bead-spring chains, based on simulations of Kopf, et al. [24], together with a fit to an eighth-order polynomial (circles), and the corresponding first logarithmic derivative $\alpha(t)$ (thin line). All chains had $N = 30$; the light chains had $m = 1$; the heavy chains had $m = 4$.

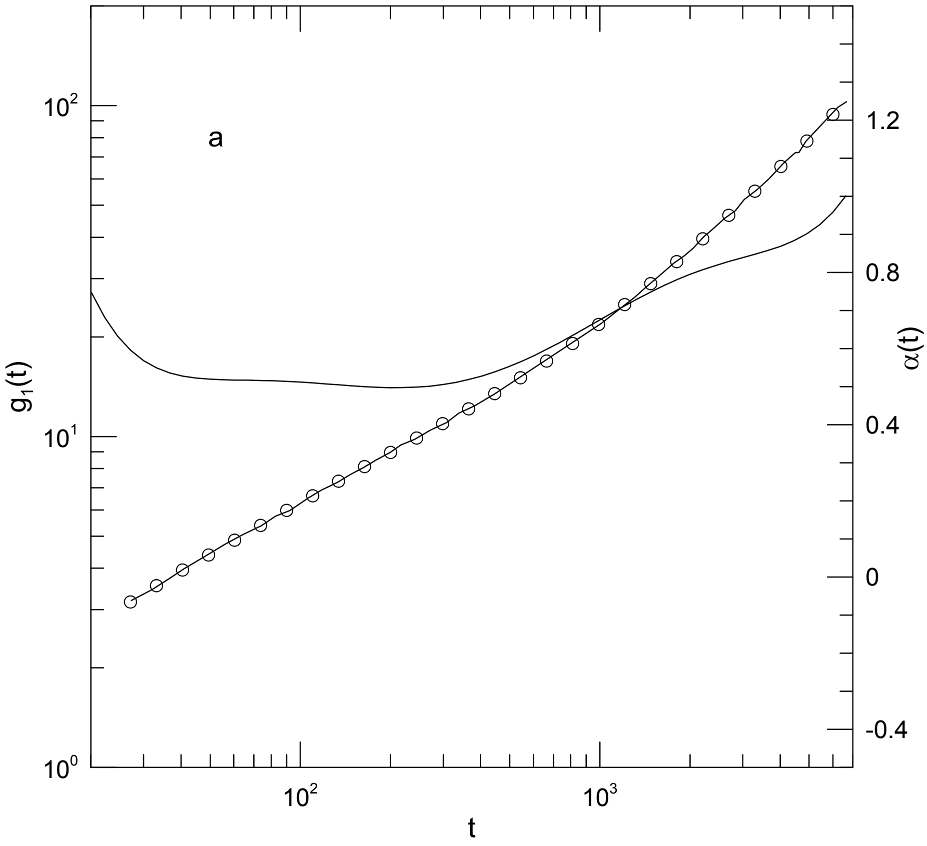

Figure S-6b)Mean-square displacement $g_3(t)$ (thick line) of the heavy chains in a 50:50 light-heavy polymer blend of Kremer-Grest bead-spring chains, based on simulations of Kopf, et al. [24], together with a fit to an eighth-order polynomial (circles), and the corresponding first logarithmic derivative $\alpha(t)$ (thin line). All chains had $N = 30$; the light chains had $m = 1$; the heavy chains had $m = 4$.

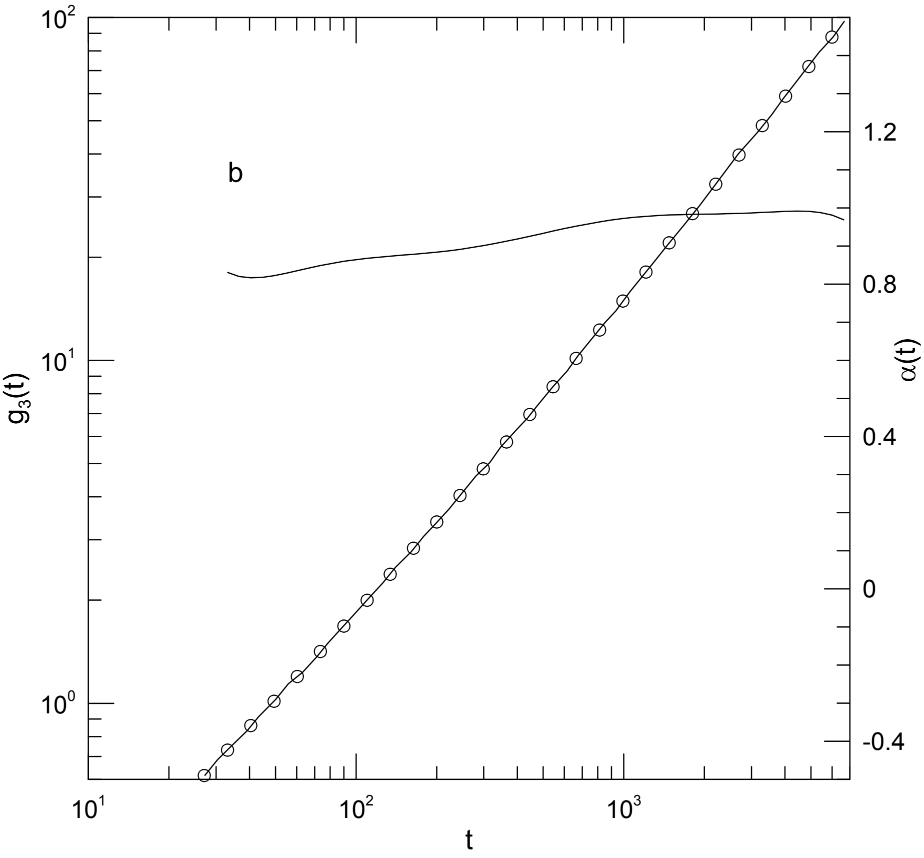

Figure S-6c) Mean-square displacement $g_1(t)$ (thick line) of the heavy chains in a 50:50 light-heavy polymer blend of Kremer-Grest bead-spring chains, based on simulations of Kopf, et al. [24], together with a fit to an eighth-order polynomial (circles), and the corresponding first logarithmic derivative $\alpha(t)$ (thin line). All chains had $N = 30$; the light chains had $m = 1$; the heavy chains had $m = 100$.

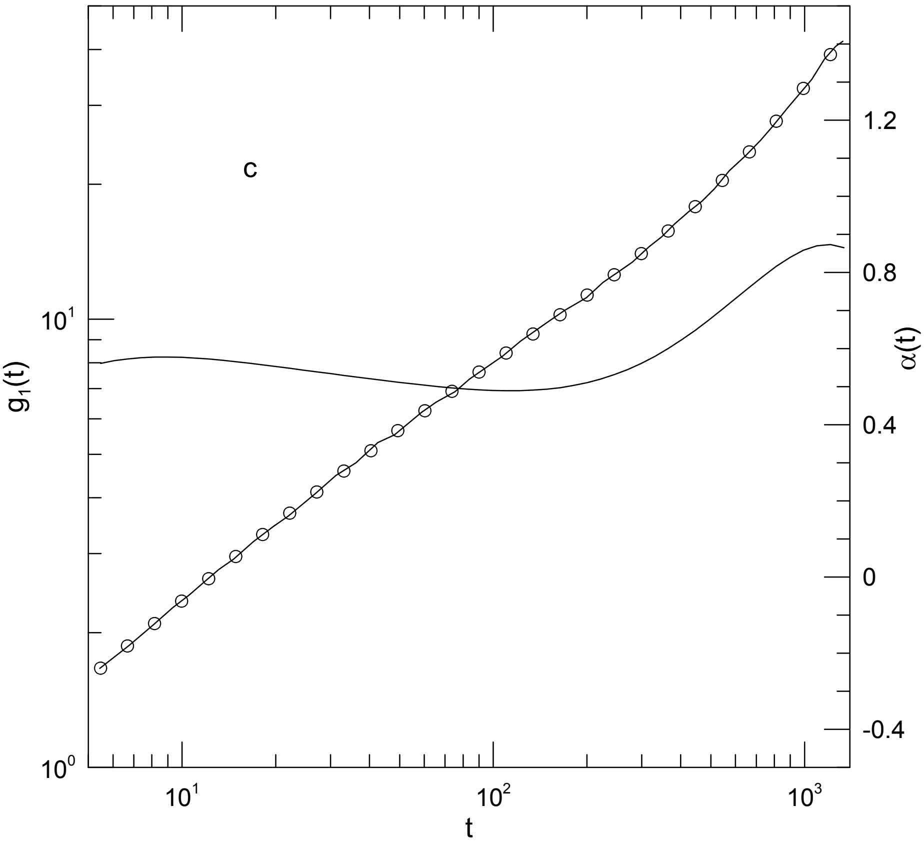

Figure S-6d) Mean-square displacement $g_3(t)$ (thick line) of the heavy chains in a 50:50 light-heavy polymer blend of Kremer-Grest bead-spring chains, based on simulations of Kopf, et al. [24], together with a fit to an eighth-order polynomial (circles), and the corresponding first logarithmic derivative $\alpha(t)$ (thin line). All chains had $N = 30$; the light chains had $m = 1$; the heavy chains had $m = 100$.

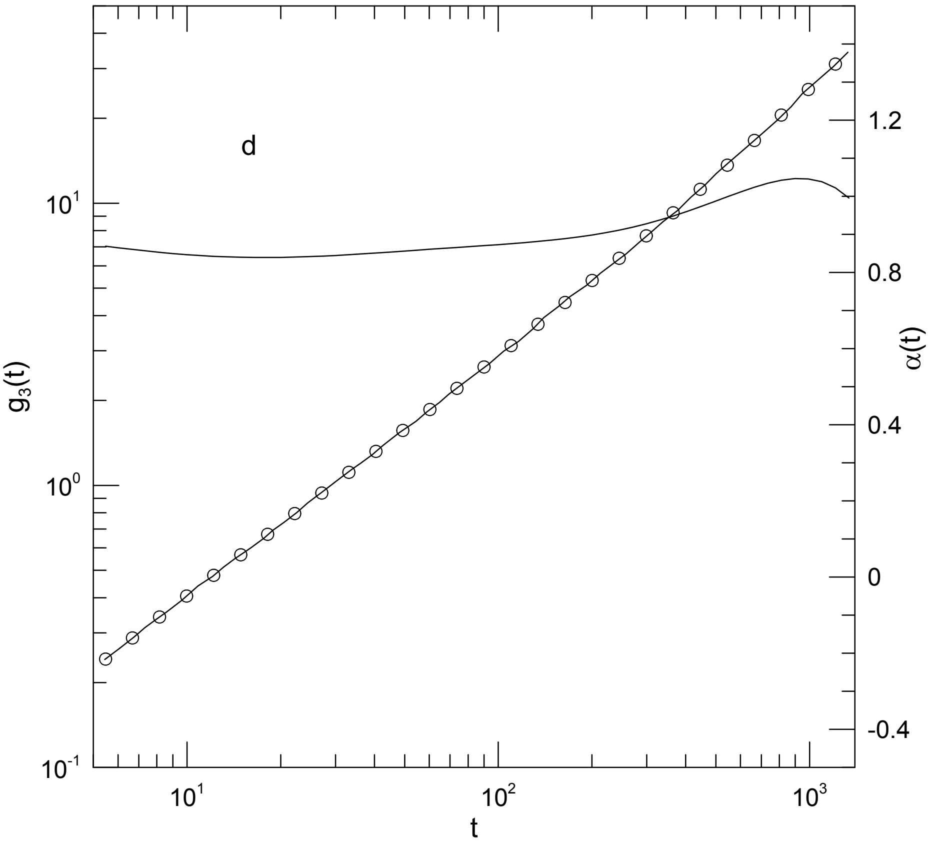

Figure S-7a) Mean-square single-bead displacement $g_1(t)$ (thick line) of 350-bead Kremer-Grest bead-spring chains dissolved in melts of (a) 25-bead bead-spring polymers, based on simulations of Wang and Larson [26], together with a fit to an eighth-order polynomial (circles), and the corresponding first logarithmic derivative $\alpha(t)$ (thin line).

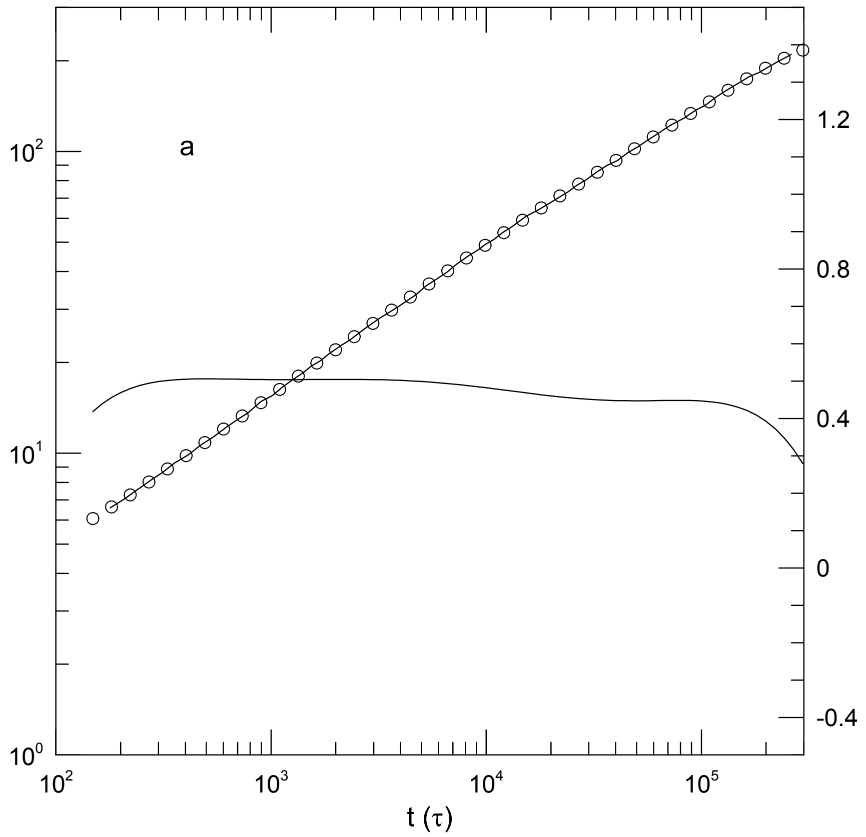

Figure S-7b) Mean-square single-bead displacement $g_1(t)$ (thick lines) of 350-bead Kremer-Grest bead-spring chains dissolved in melts of (b) 50-bead bead-spring polymers, based on simulations of Wang and Larson [26], together with a fit to an eighth-order polynomial (circles), and the corresponding first logarithmic derivative $\alpha(t)$ (thin line).

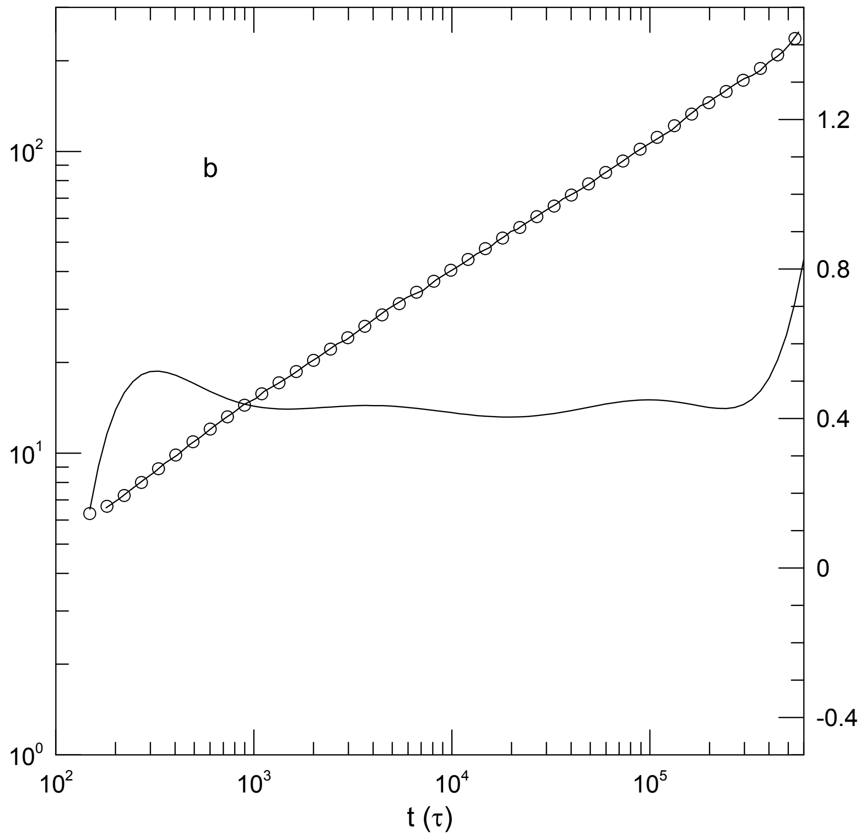

Figure S-7c) Mean-square single-bead displacement $g_1(t)$ (thick lines) of 350-bead Kremer-Grest bead-spring chains dissolved in melts of (c) 80-bead bead-spring polymers, based on simulations of Wang and Larson [26], together with a fit to an eighth-order polynomial (circles), and the corresponding first logarithmic derivative $\alpha(t)$ (thin line).

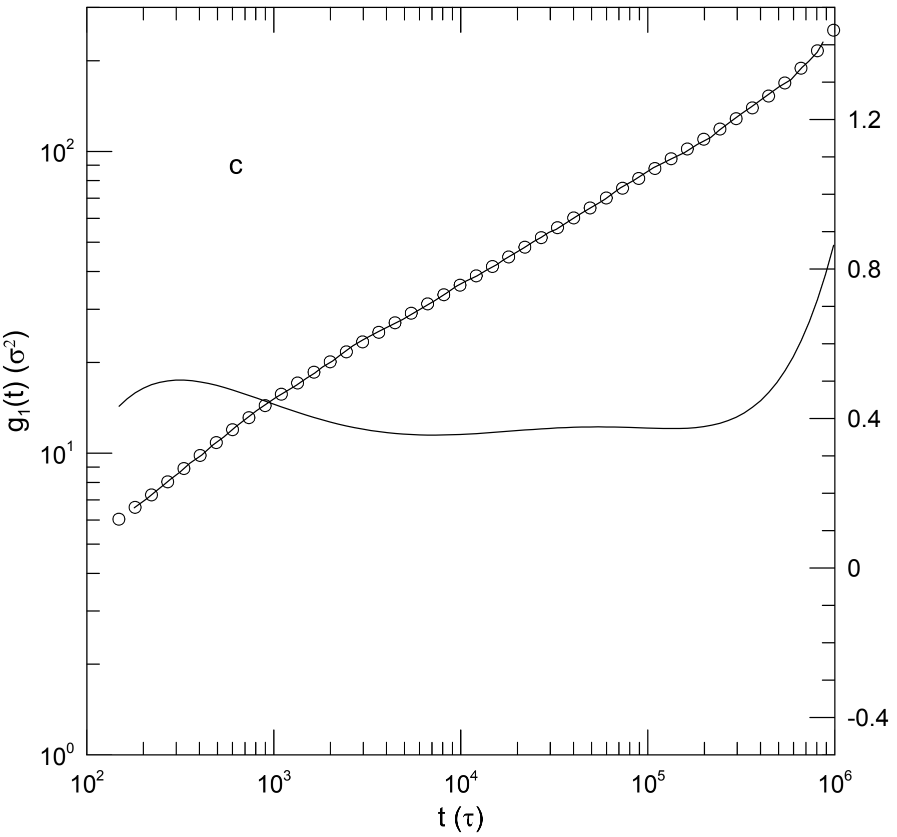

Figure S-7d) Mean-square single-bead displacement $g_1(t)$ (thick lines) of 350-bead Kremer-Grest bead-spring chains dissolved in melts of (d) 160-bead bead-spring polymers, based on simulations of Wang and Larson [26], together with a fit to an eighth-order polynomial (circles), and the corresponding first logarithmic derivative $\alpha(t)$ (thin line).

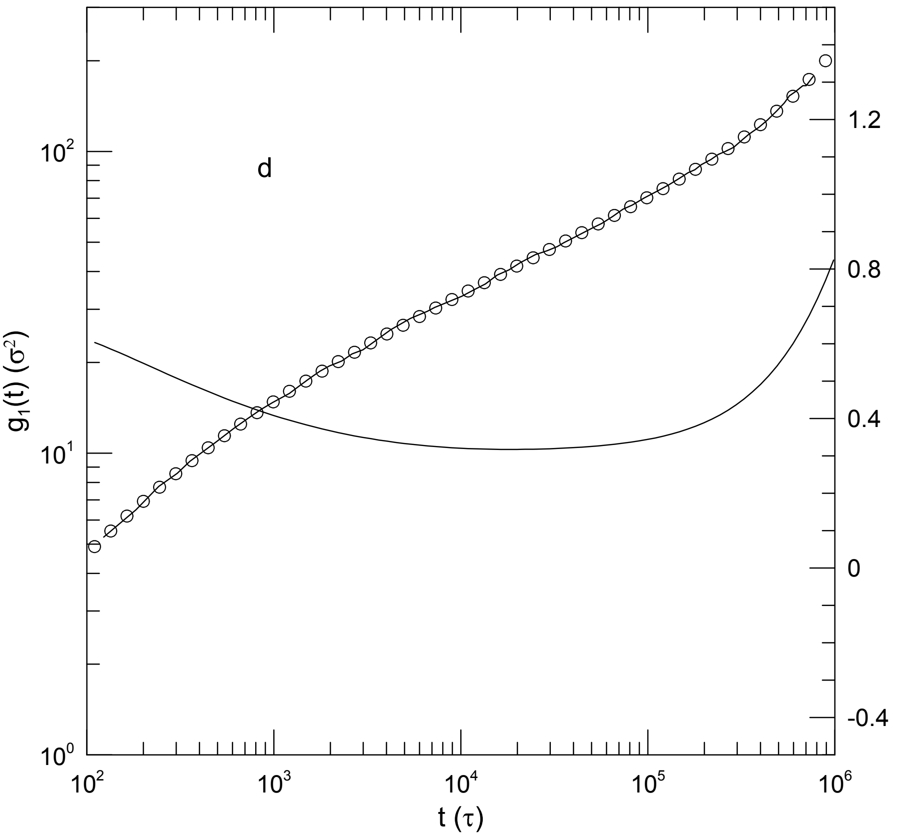

Figure S-8a) Mean-square single-bead displacement relative to chain center of mass $g_2(t)$ (thick line) of 350-bead Kremer-Grest bead-spring chains dissolved in melts of (a) 25-bead bead-spring polymers, based on simulations of Wang and Larson [26], together with a fit to an eighth-order polynomial (circles), and the corresponding first logarithmic derivative $\alpha(t)$ (thin line).

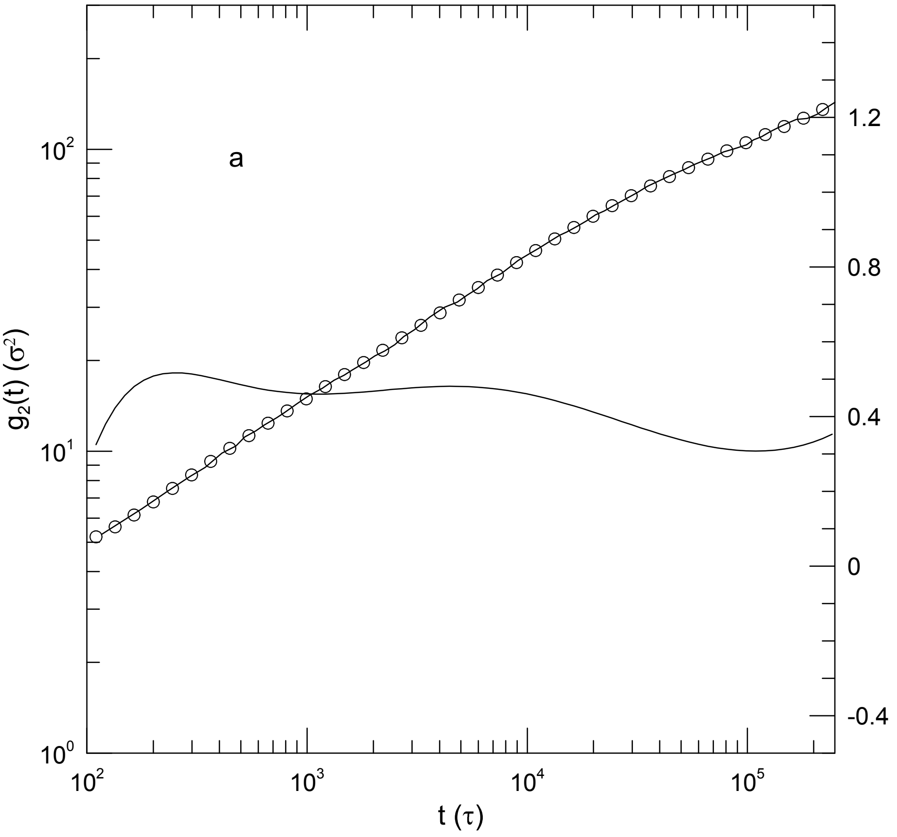

Figure S-8b) Mean-square single-bead displacement relative to chain center of mass $g_2(t)$ (thick line) of 350-bead Kremer-Grest bead-spring chains dissolved in melts of (b) 50-bead bead-spring polymers, based on simulations of Wang and Larson [26], together with a fit to an eighth-order polynomial (circles), and the corresponding first logarithmic derivative $\alpha(t)$ (thin line).

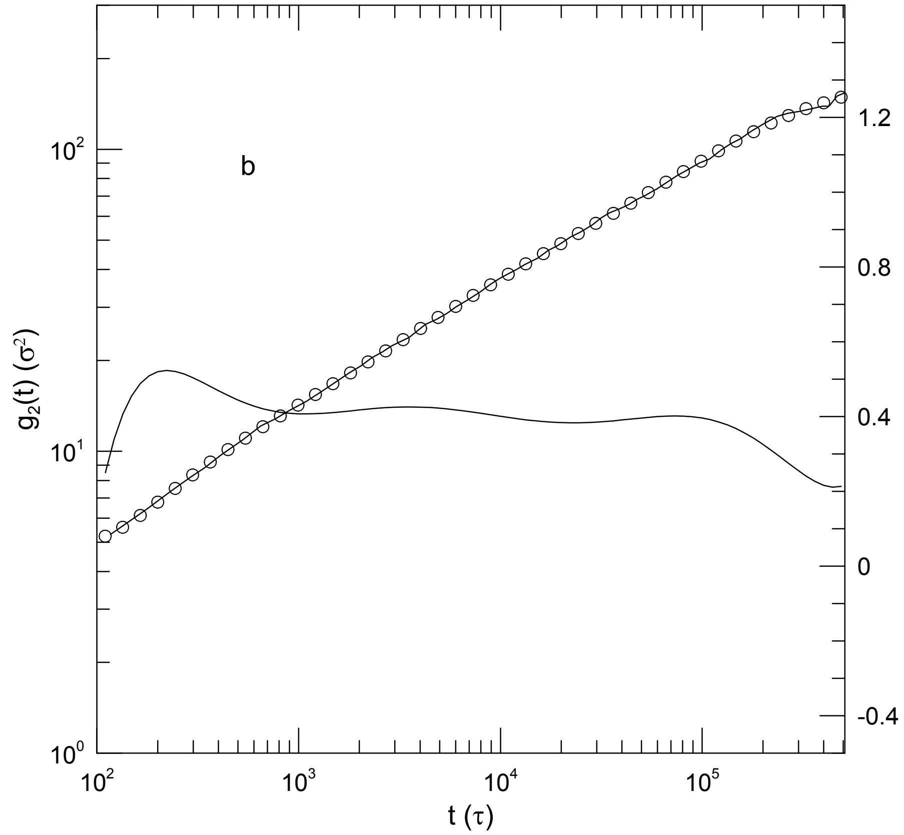

Figure S-8c) Mean-square single-bead displacement relative to chain center of mass $g_2(t)$ (thick line) of 350-bead Kremer-Grest bead-spring chains dissolved in melts of (c) 80-bead bead-spring polymers, based on simulations of Wang and Larson [26], together with a fit to an eighth-order polynomial (circles), and the corresponding first logarithmic derivative $\alpha(t)$ (thin line).

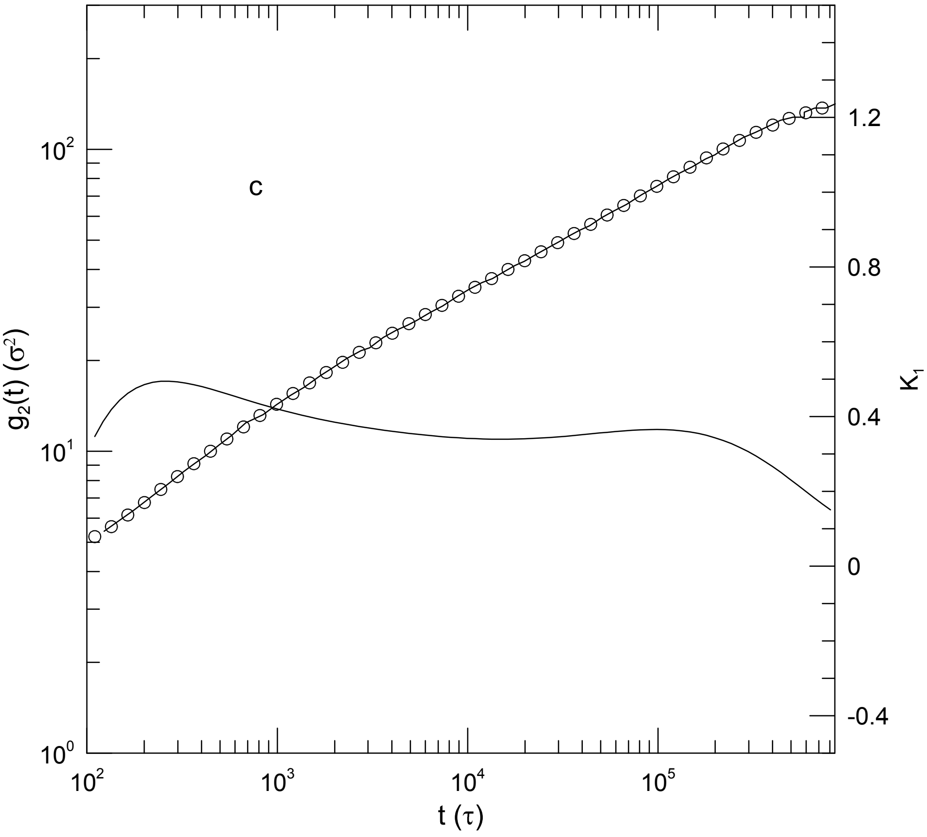

Figure S-8d) Mean-square single-bead displacement relative to chain center of mass $g_2(t)$ (thick line) of 350-bead Kremer-Grest bead-spring chains dissolved in melts of (d) 160-bead bead-spring polymers, based on simulations of Wang and Larson [26], together with a fit to an eighth-order polynomial (circles), and the corresponding first logarithmic derivative $\alpha(t)$ (thin line).

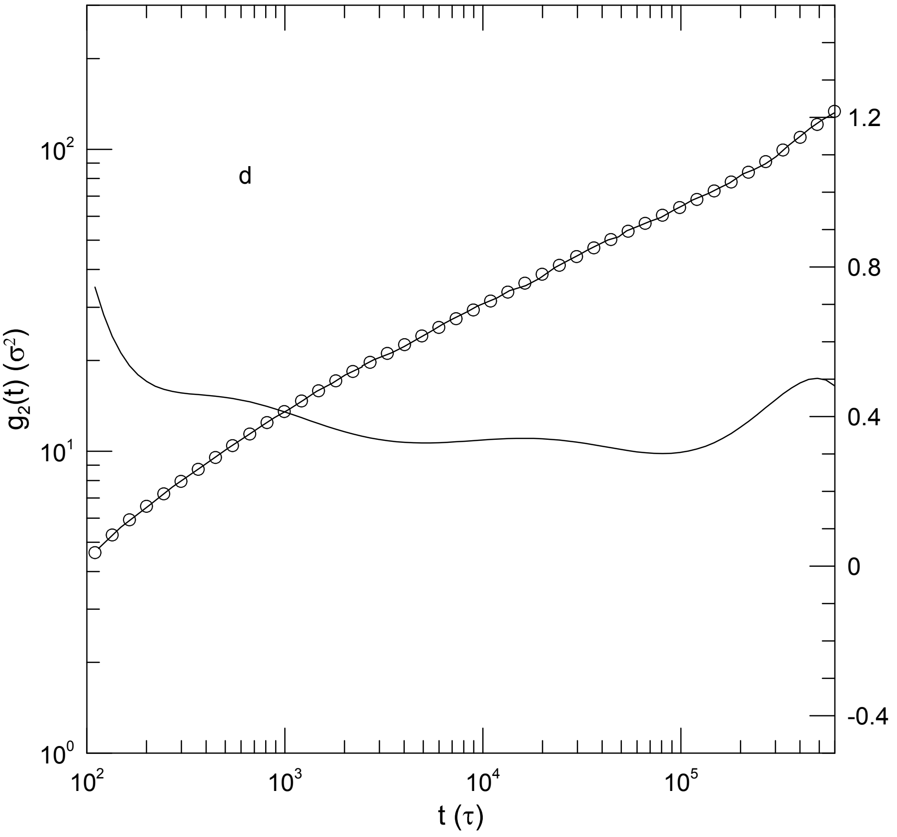

Figure S-9a) Mean-square single-bead displacement relative to chain center of mass $g_2(t)$ (thick line) of (a) 80-bead Kremer-Grest bead-spring polymers in its own melt, diluted with $\phi = 0.15$ volume fraction of a 350-bead polymer having the same potential energy parameters, based on simulations of Wang and Larson [26], together with a fit to an eighth-order polynomial (circles), and the corresponding first logarithmic derivative $\alpha(t)$ (thin line).

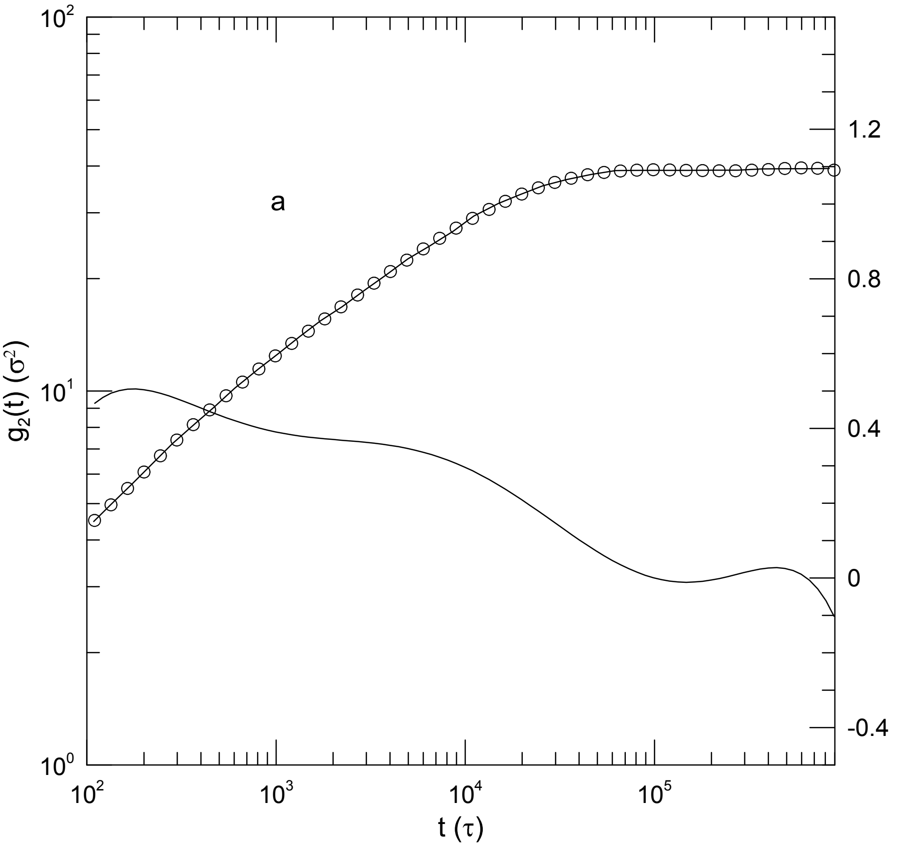

Figure S-9b) Mean-square single-bead displacement relative to chain centers of mass $g_2(t)$ (thick line) of (b) 50-bead Kremer-Grest bead-spring polymers in its own melt, diluted with $\phi = 0.15$ volume fraction of a 350-bead polymer having the same potential energy parameters, based on simulations of Wang and Larson [26], together with a fit to an eighth-order polynomial (circles), and the corresponding first logarithmic derivative $\alpha(t)$ (thin line).

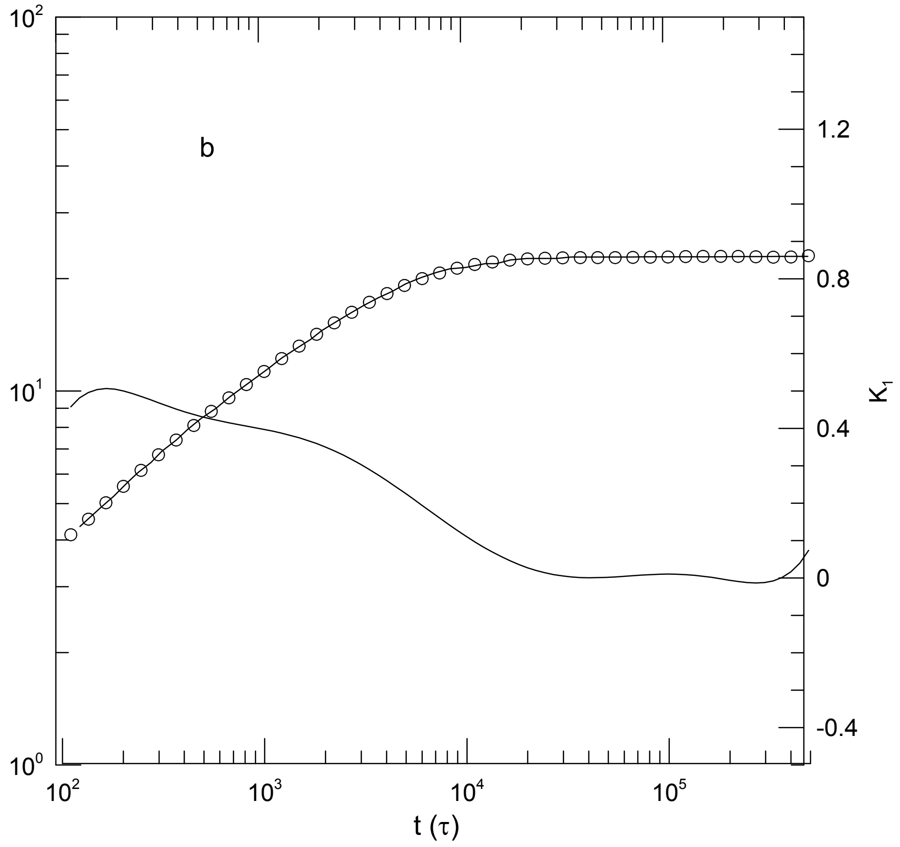

Figure S-9c) Mean-square single-bead displacement relative to chain centers of mass $g_2(t)$ (thick line) of (c) 25-bead Kremer-Grest bead-spring polymers in its own melt, diluted with $\phi = 0.15$ volume fraction of a 350-bead polymer having the same potential energy parameters, based on simulations of Wang and Larson [26], together with a fit to an eighth-order polynomial (circles), and the corresponding first logarithmic derivative $\alpha(t)$ (thin line).

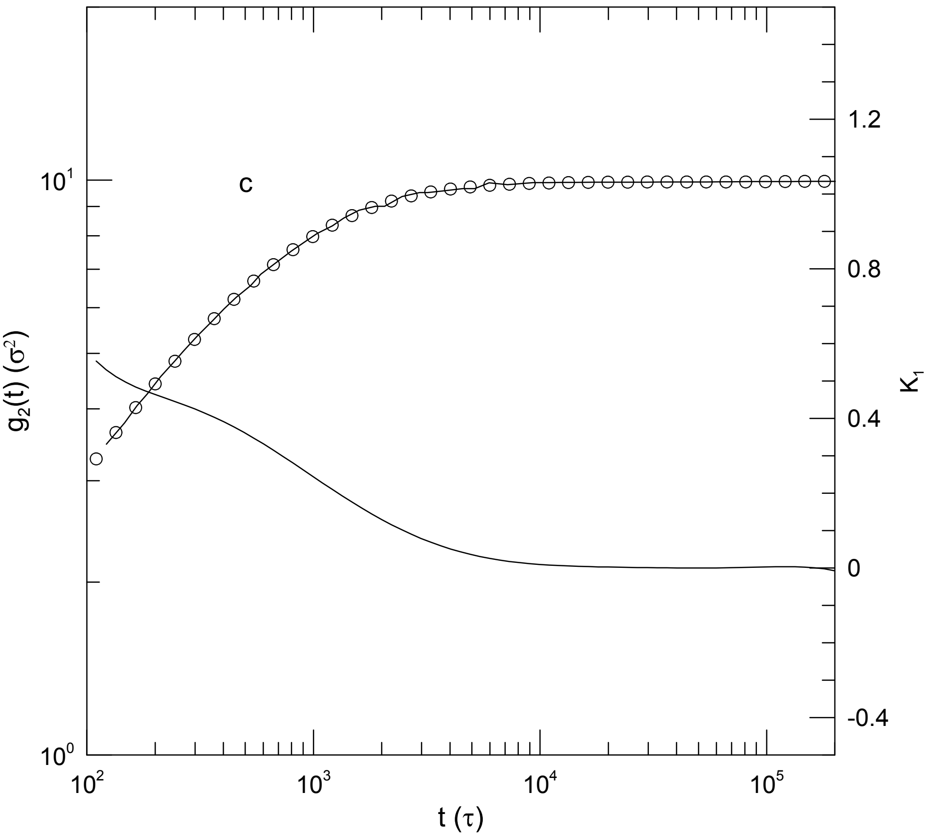

Figure S-10a) Mean-square center-of-mass displacement $g_3(t)$ (thick line) of 350-bead Kremer-Grest bead-spring chains dissolved in melts of (a) 25-bead bead-spring polymers, based on simulations of Wang and Larson [26], together with a fit an to eighth-order polynomials (circles), and the corresponding first logarithmic derivative $\alpha(t)$ (thin line).

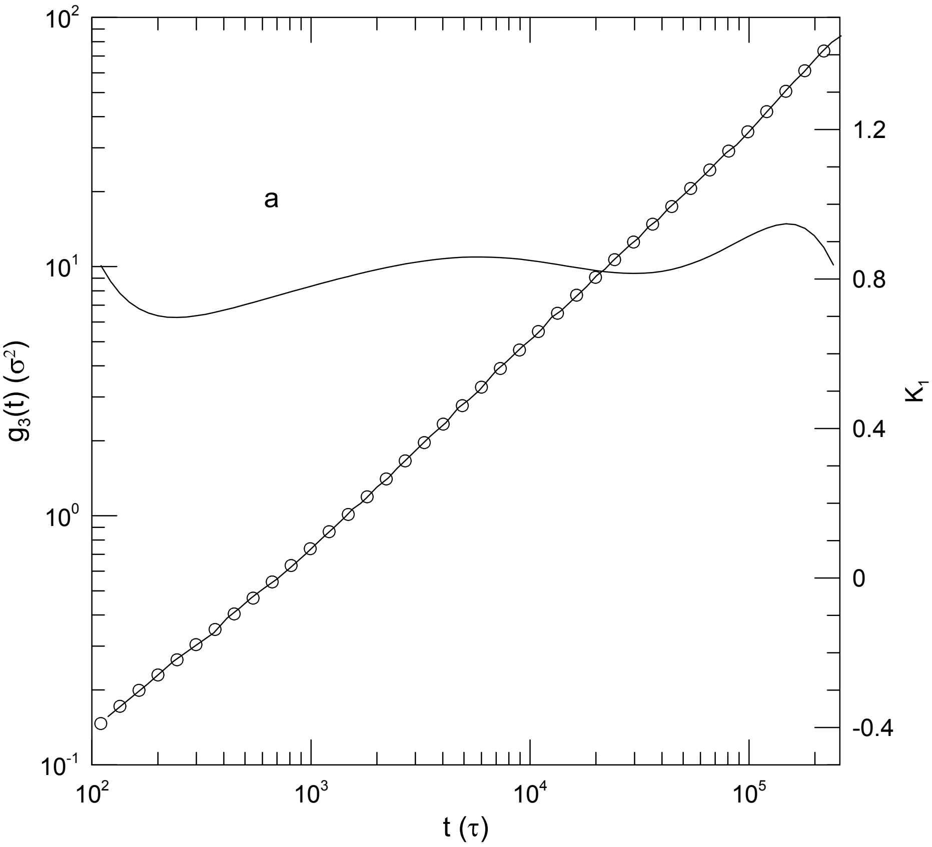

Figure S-10b) Mean-square center-of-mass displacement $g_3(t)$ (thick line) of 350-bead Kremer-Grest bead-spring chains dissolved in melts of (b) 50-bead bead-spring polymers, based on simulations of Wang and Larson [26], together with a fit to an eighth-order polynomial (circles), and the corresponding first logarithmic derivative $\alpha(t)$ (thin line).

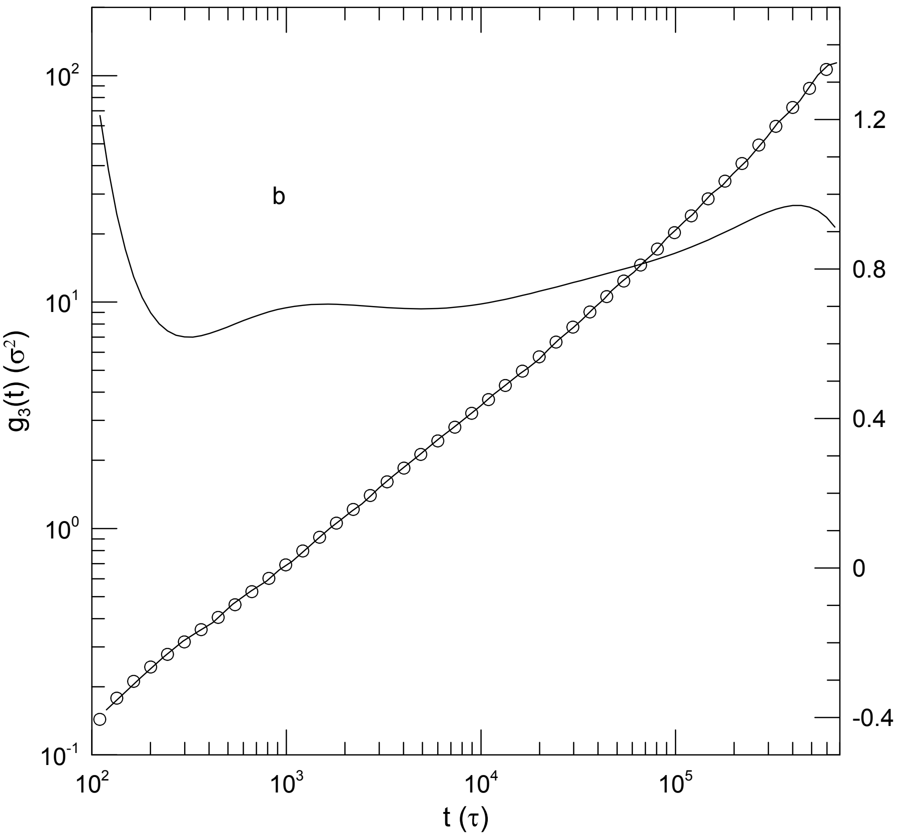

Figure S-10c) Mean-square center-of-mass displacement $g_3(t)$ (thick line) of 350-bead Kremer-Grest bead-spring chains dissolved in melts of (c) 80-bead bead-spring polymers, based on simulations of Wang and Larson [26], together with a fit to an eighth-order polynomial (circles), and the corresponding first logarithmic derivative $\alpha(t)$ (thin line).

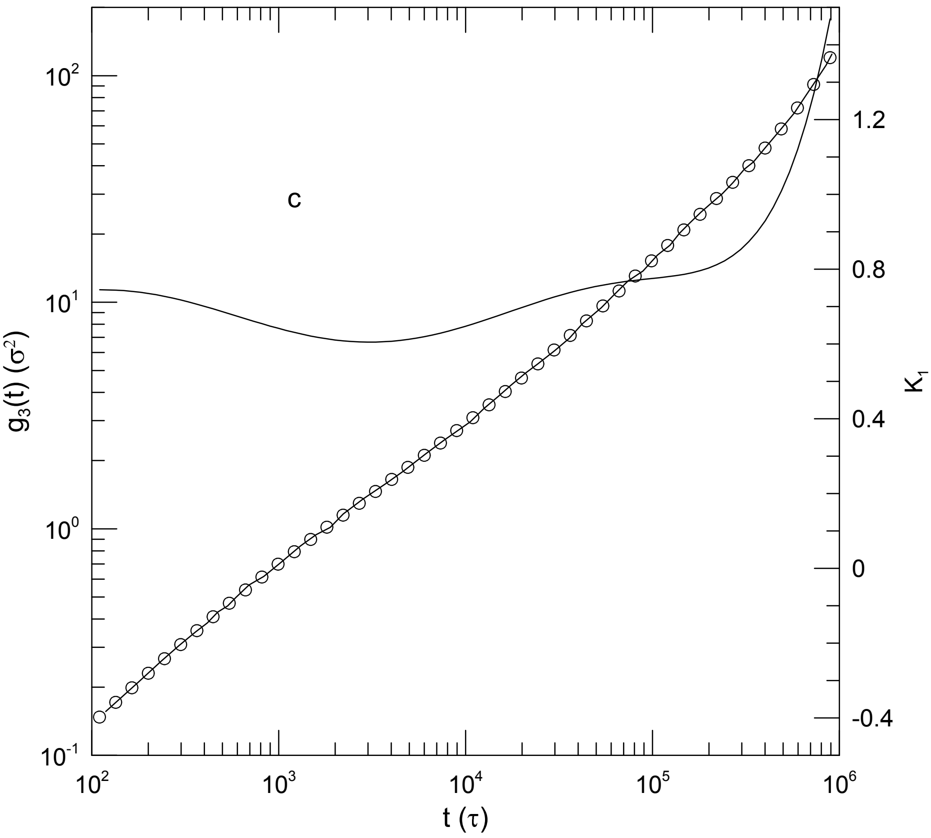

Figure S-10d) Mean-square center-of-mass displacement $g_3(t)$ (thick line) of 350-bead Kremer-Grest bead-spring chains dissolved in melts of d) 160-bead bead-spring polymers, based on simulations of Wang and Larson [26], together with a fit to an eighth-order polynomials (circles), and the corresponding first logarithmic derivative $\alpha(t)$ (thin line).

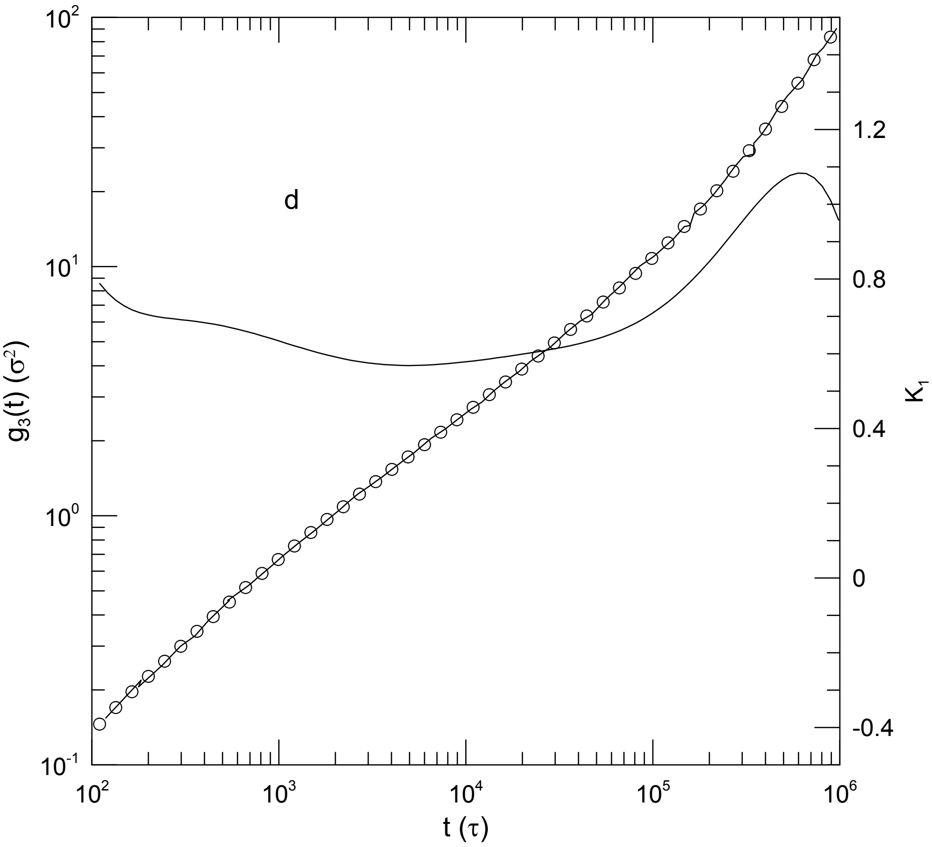

Figure S-11a) Mean-square displacement (a) $g_1(t)$ (thick line) of monodisperse melts of 350-bead Kremer-Grest bead-spring chains, based on simulations of Wang and Larson [26], together with a fit to an eighth-order polynomials (circles), and the corresponding first logarithmic derivative $\alpha(t)$ (thin line).

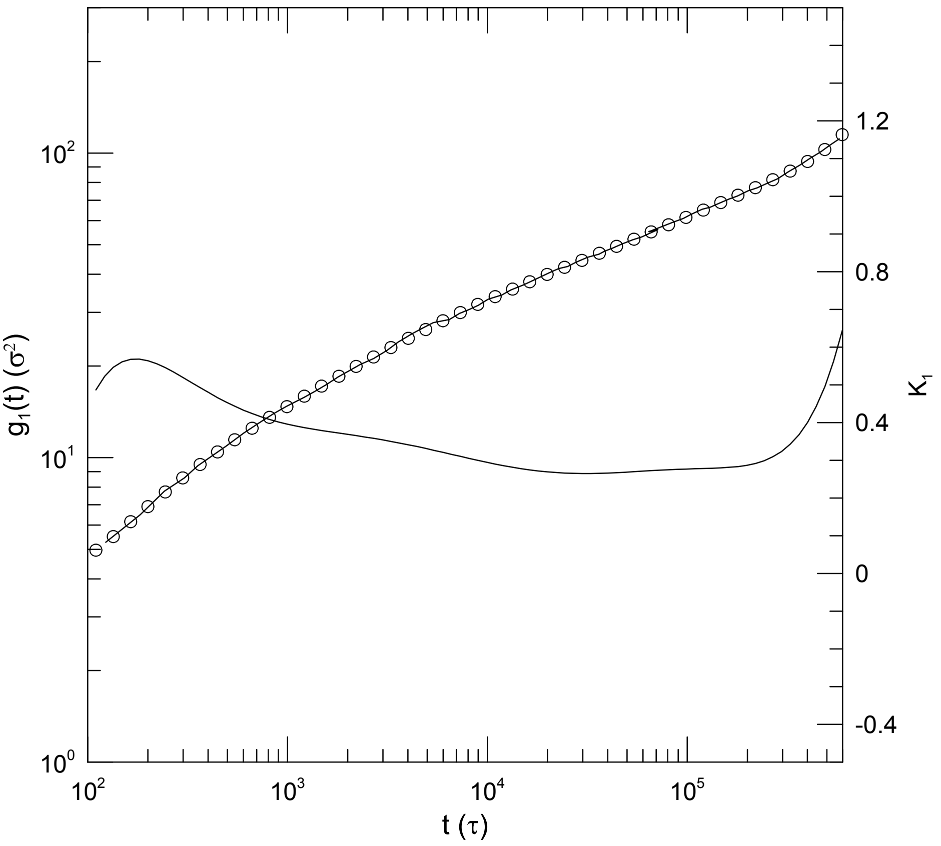

Figure S-11b) Mean-square displacement (b) $g_3(t)$ (thick line) of monodisperse melt of 350-bead Kremer-Grest bead-spring chains, based on simulations of Wang and Larson [26], together with a fit to an eighth-order polynomial (circles), and the corresponding first logarithmic derivative $\alpha(t)$ (thin line).

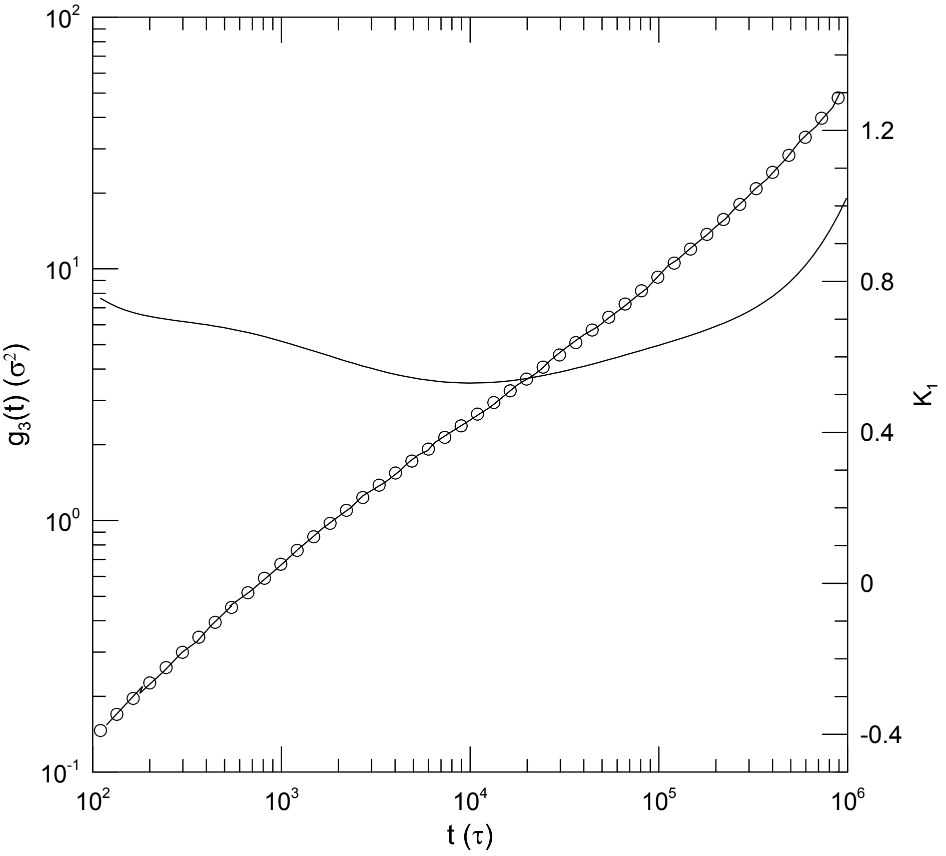

Figure S-12a) Mean-square central bead displacement $g_1(t)$ (thick line) of melts of monodisperse Kremer-Grest bead-spring chains, based on simulations of Adeyemi, et al. [25], together with a fit to an eighth-order polynomial (circles) and the corresponding first logarithmic derivative $\alpha(t)$ (thin line). Chains contained (a) 25 beads. Note the long-time behavior, namely a decrease in $\alpha(t)$ at long times. The fitted curve (circles) has been extrapolated beyond the limits of the data; the behavior of $\alpha(t)$ becomes anomalous.

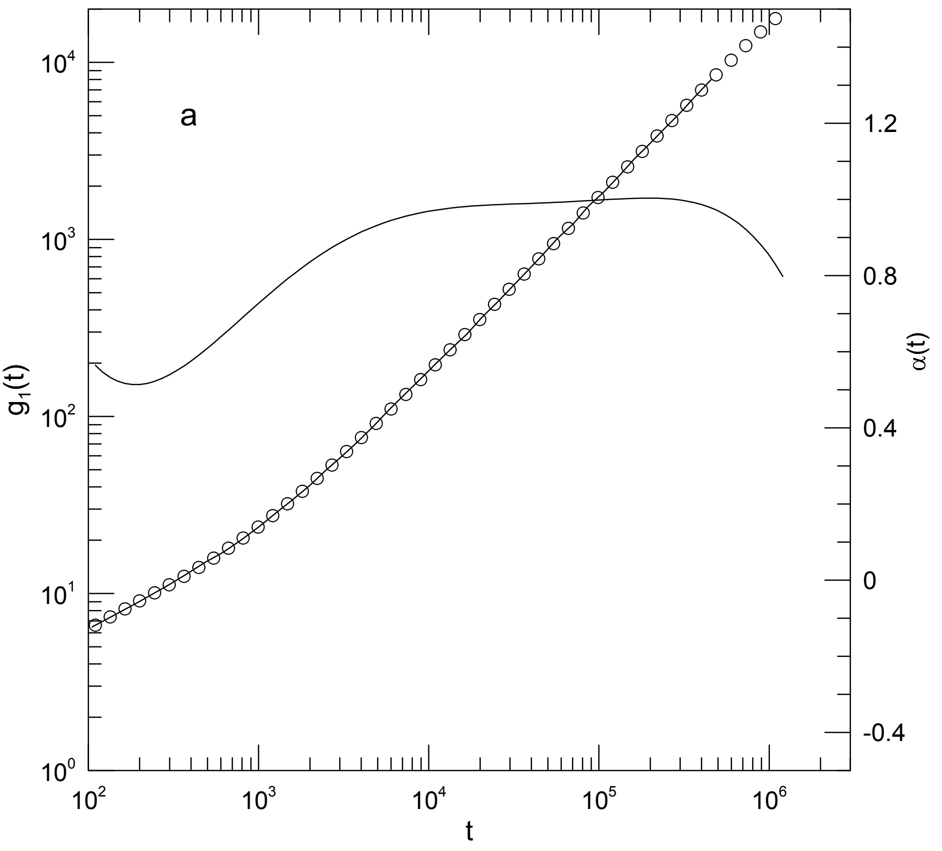

Figure S-12b) Mean-square central bead displacement $g_1(t)$ (thick line) of melts of monodisperse Kremer-Grest bead-spring chains, based on simulations of Adeyemi, et al. [25], together with a fit to an eighth-order polynomial (circles) and the corresponding first logarithmic derivative $\alpha(t)$ (thin line). Chains contained (b) 50 beads.

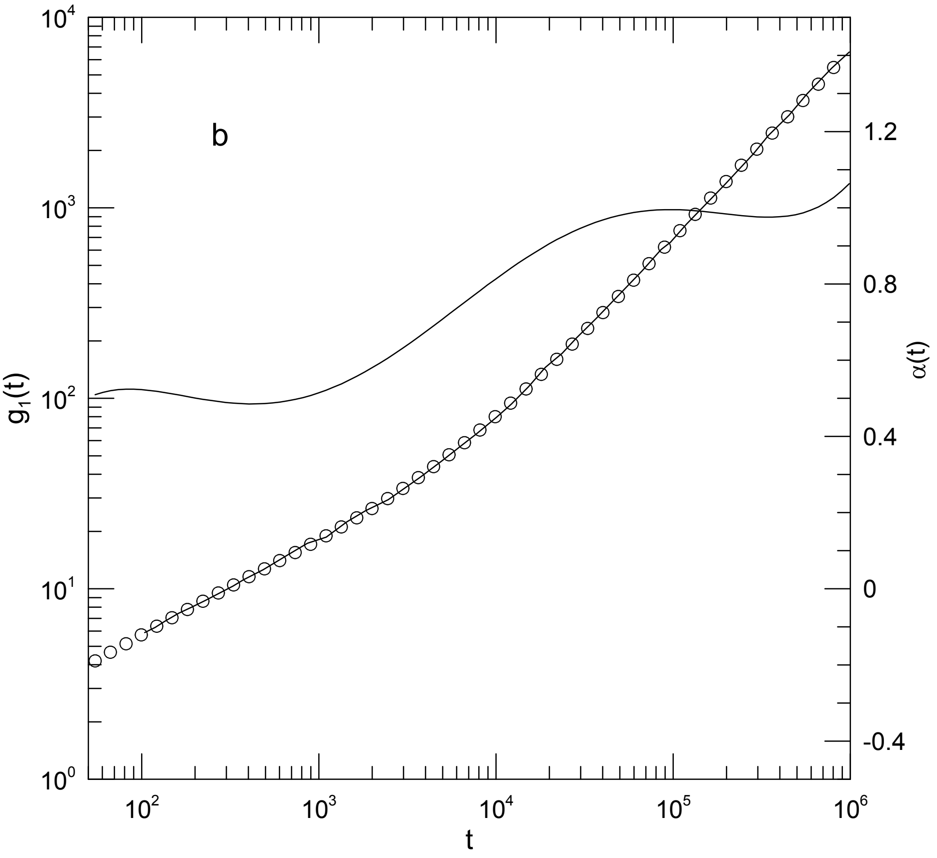

Figure S-12c) Mean-square central bead displacement $g_1(t)$ (thick line) of melts of monodisperse Kremer-Grest bead-spring chains, based on simulations of Adeyemi, et al. [25], together with a fit to an eighth-order polynomial (circles) and the corresponding first logarithmic derivative $\alpha(t)$ (thin line). Chains contained (c) 100 beads.

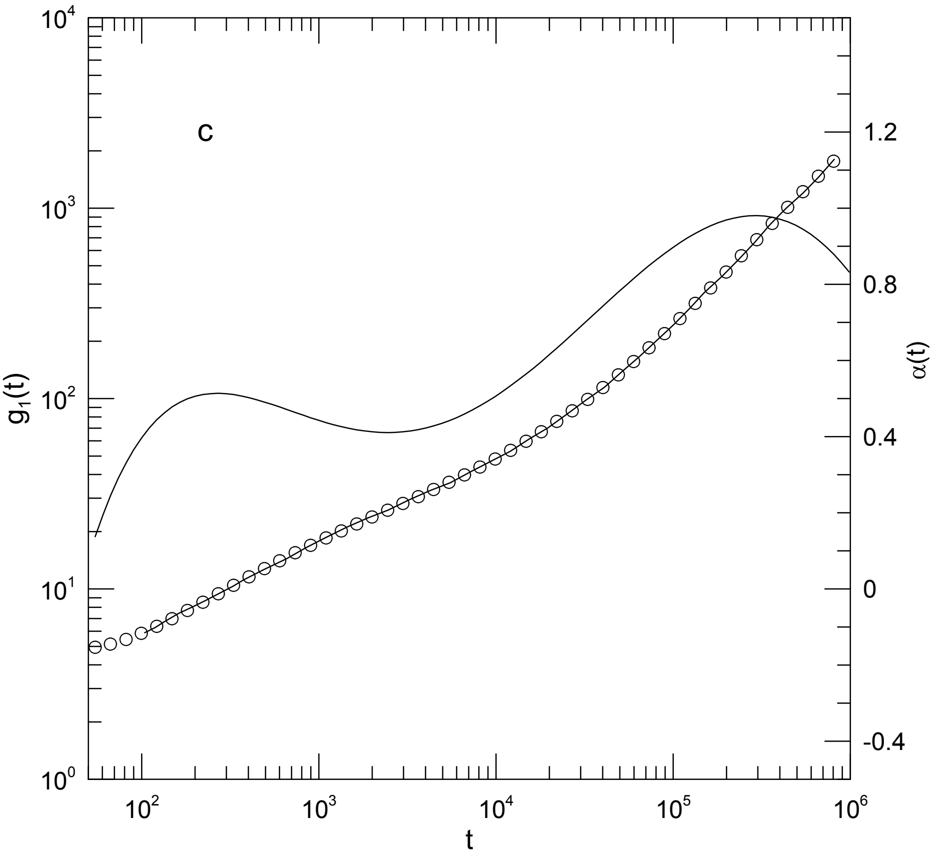

Figure S-12d) Mean-square central bead displacement $g_1(t)$ (thick line) of melts of monodisperse Kremer-Grest bead-spring chains, based on simulations of Adeyemi, et al. [25], together with a fit to an eighth-order polynomial (circles) and the corresponding first logarithmic derivative $\alpha(t)$ (thin line). Chains contained (d) 350 beads.

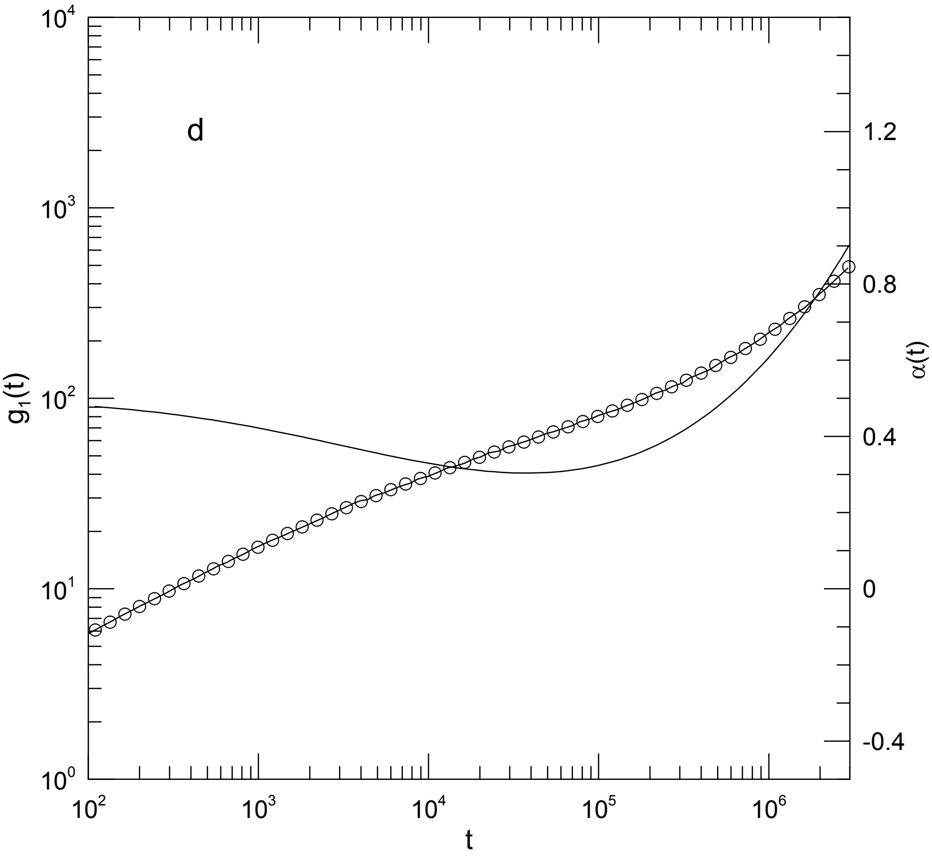

Figure S-13a) Mean-square center-of-mass displacement $g_3(t)$ (thick line) of 350-bead Kremer-Grest bead-spring chains in its own melt, based on simulations of Adeyemi, et al. [25], together with a fit to an eighth-order polynomial (circles), and the corresponding first logarithmic derivative $\alpha(t)$ (thin line).

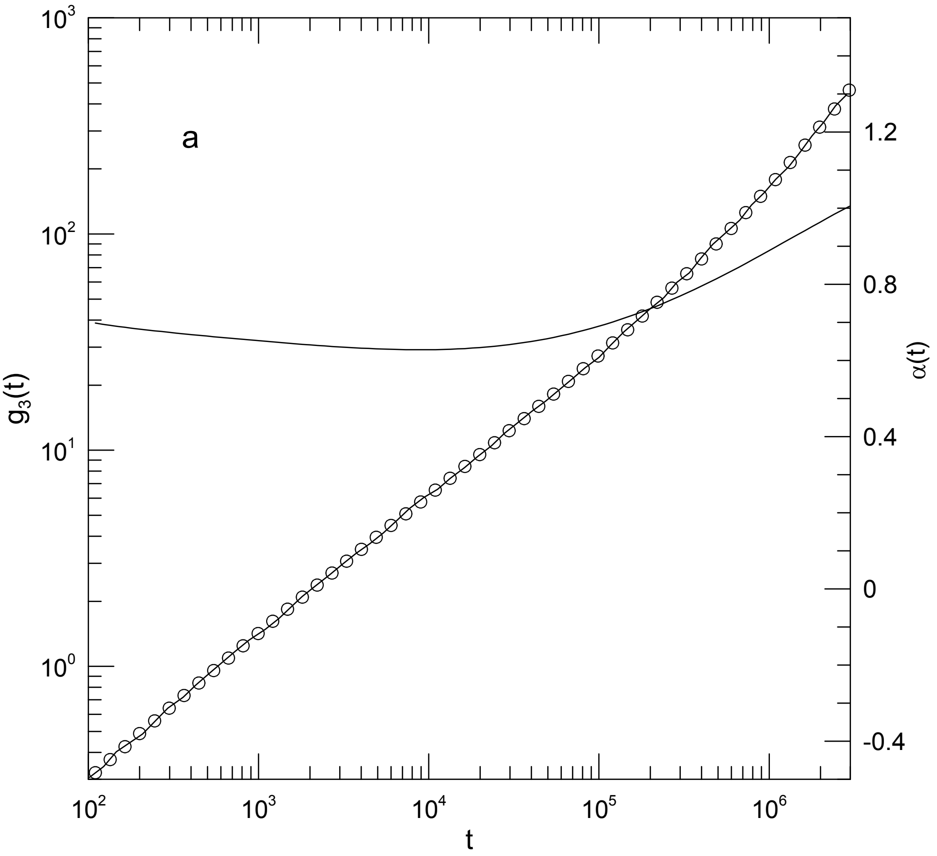

Figure S-13b) Mean-square center-of-mass displacement $g_3(t)$ (thick line) of 350-bead Kremer-Grest bead-spring chains, based on simulations of Adeyemi, et al. [25], together with a fits to an eighth-order polynomial (circles), and the corresponding first logarithmic derivative $\alpha(t)$ (thin line). Figure refers to a blends with 0.7 volume fraction of the 350-bead polymer and 0.3 volume fraction of the (b) 100-bead polymer.

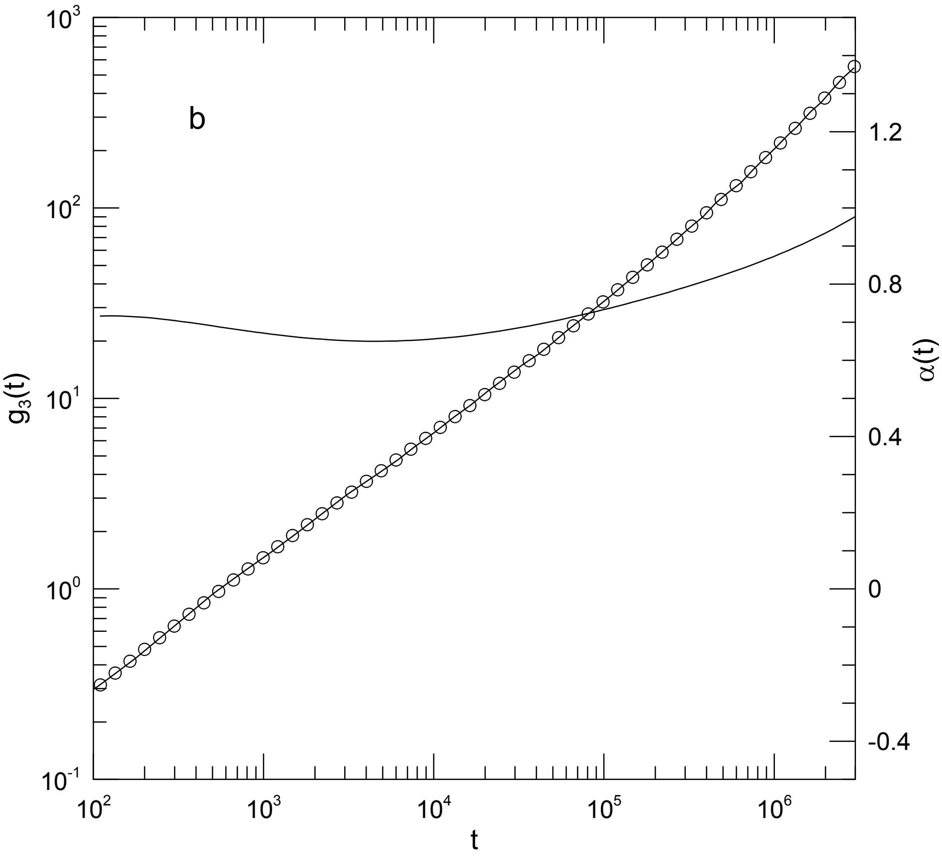

Figure S-13c) Mean-square center-of-mass displacement $g_3(t)$ (thick line) of 350-bead Kremer-Grest bead-spring chains, based on simulations of Adeyemi, et al. [25], together with a fits to an eighth-order polynomial (circles), and the corresponding first logarithmic derivative $\alpha(t)$ (thin line). Figure refers to a blends with 0.7 volume fraction of the 350-bead polymer and 0.3 volume fraction of the (c) 50-bead polymer.

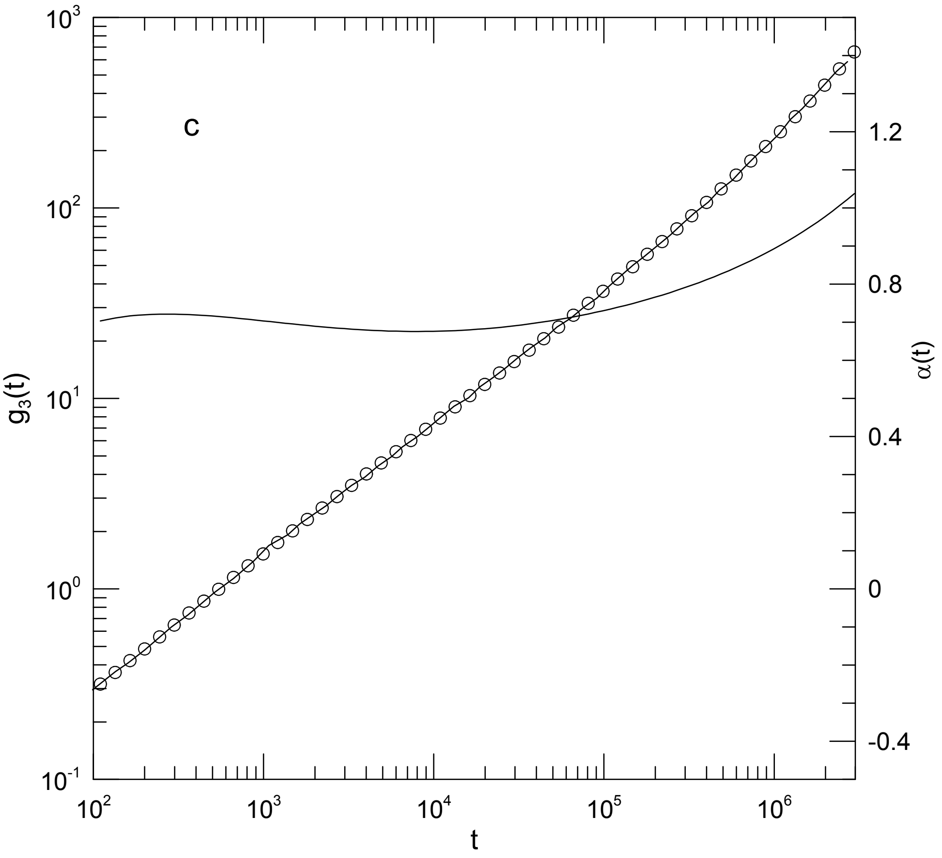

Figure S-13d) Mean-square center-of-mass displacement $g_3(t)$ (thick line) of 350-bead Kremer-Grest bead-spring chains, based on simulations of Adeyemi, et al. [25], together with a fits to an eighth-order polynomial (circles), and the corresponding first logarithmic derivative $\alpha(t)$ (thin line). Figure refers to a blends with 0.7 volume fraction of the 350-bead polymer and 0.3 volume fraction of the (d) 25-bead polymer.

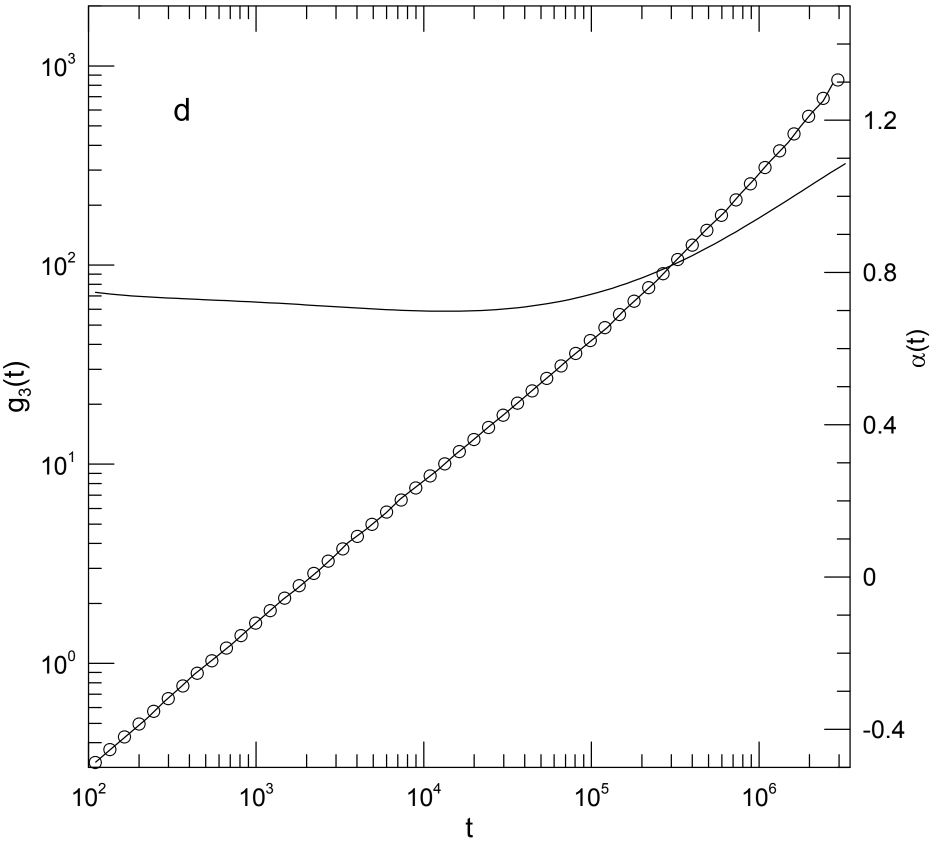

Figure S-14a) Mean-square center-of-mass displacement $g_3(t)$ (thick line) of 350-bead Kremer-Grest bead-spring chains, based on simulations of Adeyemi, et al. [25], together with a fits to an eighth-order polynomial (circles), and the corresponding first logarithmic derivative $\alpha(t)$ (thin line). The blend here is 0.3 volume fraction of the 350-bead polymer and 0.7 volume fraction of the (a) 100-bead polymer.

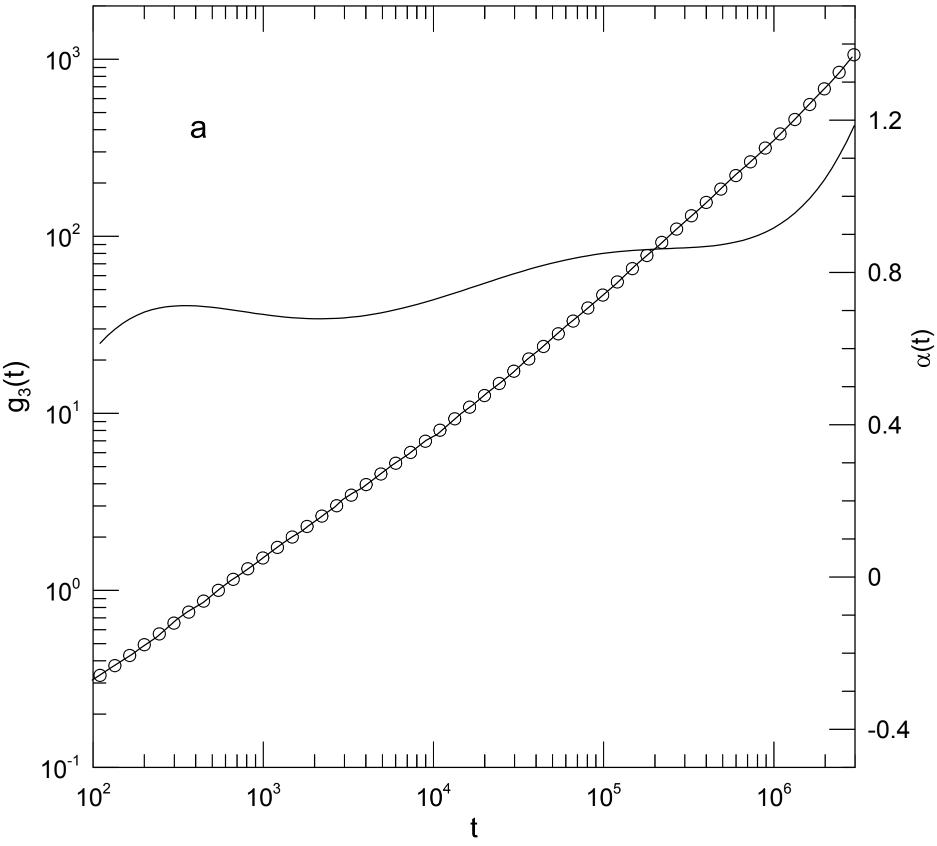

Figure S-14b) Mean-square center-of-mass displacement $g_3(t)$ (thick line) of 350-bead Kremer-Grest bead-spring chains, based on simulations of Adeyemi, et al. [25], together with a fits to an eighth-order polynomial (circles), and the corresponding first logarithmic derivative $\alpha(t)$ (thin line). The blend here is 0.3 volume fraction of the 350-bead polymer and 0.7 volume fraction of the (b) 50-bead polymer.

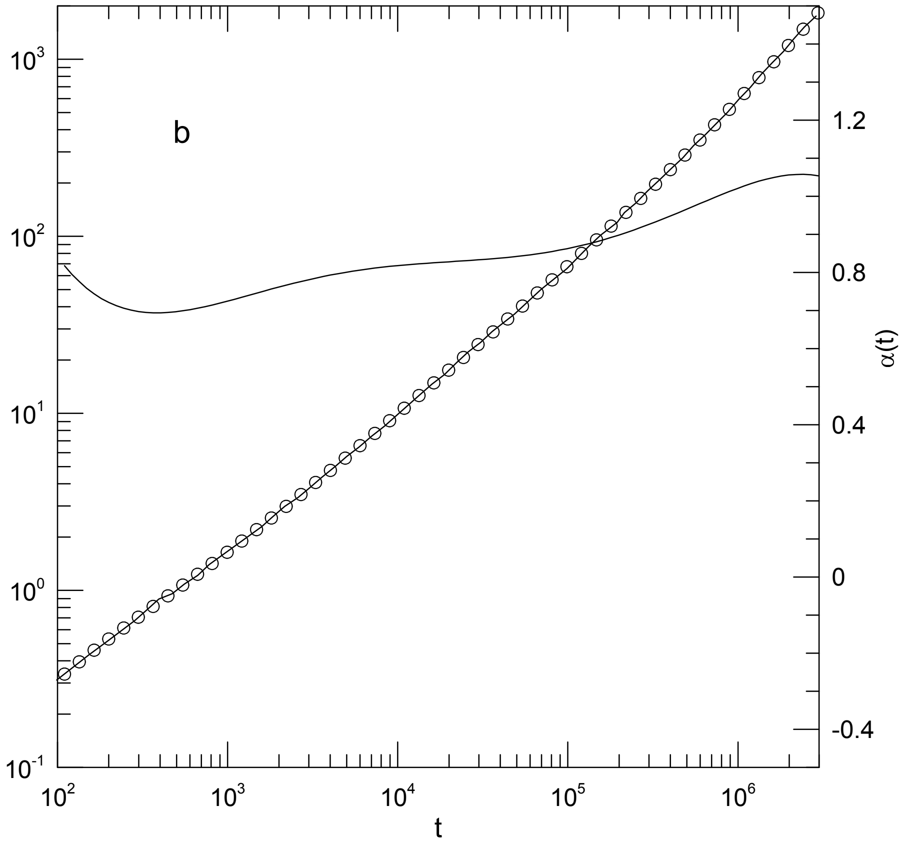

Figure S-14c) Mean-square center-of-mass displacement $g_3(t)$ (thick line) of 350-bead Kremer-Grest bead-spring chains, based on simulations of Adeyemi, et al. [25], together with a fits to an eighth-order polynomial (circles), and the corresponding first logarithmic derivative $\alpha(t)$ (thin line). The blend here is 0.3 volume fraction of the 350-bead polymer and 0.7 volume fraction of the (c) 25-bead polymer.

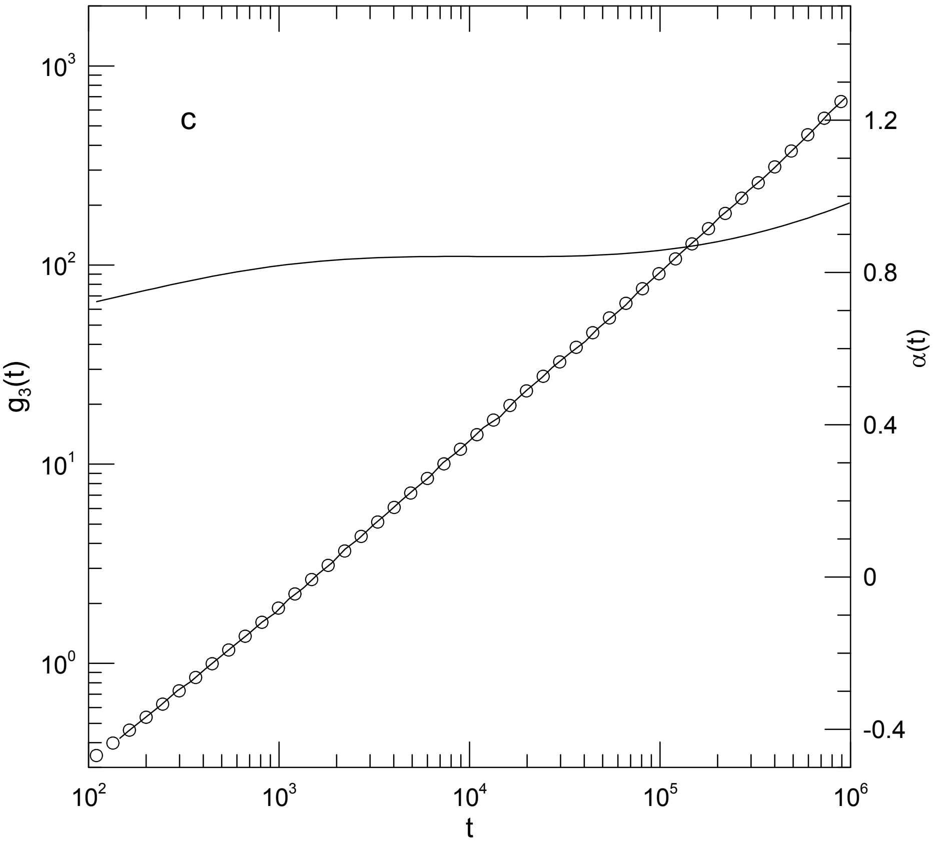

The Supplemental Material reproduces at full scale the text figures, so that the quality of the fits may more easily be appreciated. Figure and Citation numbers are the same as in the main text, so that, e.g., Figure S-1a of the Supplemental Material is the same as Figure 1a of the text.
